# Supplementary material for: Ameliorative Effects of Flavonoids from Platycodon grandiflorus Aerial Parts on Alloxan-Induced Pancreatic Islet Damage in Zebrafish
Source: Nutrients. 2023 Apr 6;15(7):1798. doi: 10.3390/nu15071798 (PMC10096680; doi:10.3390/nu15071798)
Supplement: Supplementary file 1 [file nutrients-15-01798-s001.zip › nutrients-2282467-supplementary.pdf]

SUPPORTING INFORMATION

Ameliorative Effects of Flavonoids from *Platycodon grandiflorus* Aerial Part on Alloxan-Induced Pancreatic Islet Damage in Zebrafish

Youn Hee Nam [1,†], Eun Bin Kim [2,†], Ji Eun Kang [2], Ju Su Kim [2], Yukyoung Jeon [2], Sung Woo Shin [1], Tong Ho Kang [1,*], and Jong Hwan Kwak [2,*]

[1] Department of Oriental Medicine Biotechnology, College of Life Sciences and Graduate School of Biotechnology, Kyung Hee University, Global Campus, Gyeonggi 17104, Korea

[2] School of Pharmacy, Sungkyunkwan University, Suwon, Gyeonggi-do 16419, Republic of Korea

* Correspondence: panjae@khu.ac.kr (T.H.K.); jhkwak@skku.edu (J.H.K.); Tel.: +82-31-201-3862 (T.H.K.); +82-31-290-7745(J.H.K.); Fax: +82-303-0300-0030 (T.H.K.); +82-31-292-8800 (J.H.K.)

† These two authors contributed equally to this work.

List of Contents

Figure S1. ESIMS (positive ion mode) spectrum of compound **1**.

Figure S2. FABMS (positive ion mode) spectrum of compound **1**.

Figure S3. HRFABMS (positive ion mode) spectrum of compound **1**.

Figure S4. ^{1}H NMR spectrum of compound **1** (CD_3OD, 700 MHz).

Figure S5. ^{13}C NMR spectrum of compound **1** (CD_3OD, 176 MHz).

Figure S6. ^{1}H-^{1}H COSY spectrum of compound **1** (CD_3OD, 700 MHz).

Figure S7. HSQC spectrum of compound **1** (CD_3OD, 700 MHz).

Figure S8. HMBC spectrum of compound **1** (CD_3OD, 700 MHz).

Figure S9. ESIMS (positive ion mode) spectrum of compound **2**.

Figure S10. FABMS (positive ion mode) spectrum of compound **2**.

Figure S11. HRFABMS (positive ion mode) spectrum of compound **2**.

Figure S12. ^{1}H NMR spectrum of compound **2** (DMSO-d_6, 700 MHz).

Figure S13. ^{13}C NMR spectrum of compound **2** (DMSO-d_6, 176 MHz).

Figure S14. ^{1}H-^{1}H COSY spectrum of compound **2** (DMSO-d_6, 700 MHz).

Figure S15. HSQC spectrum of compound **2** (DMSO-d_6, 700 MHz).

Figure S16. HMBC spectrum of compound **2** (DMSO-d_6, 700 MHz).

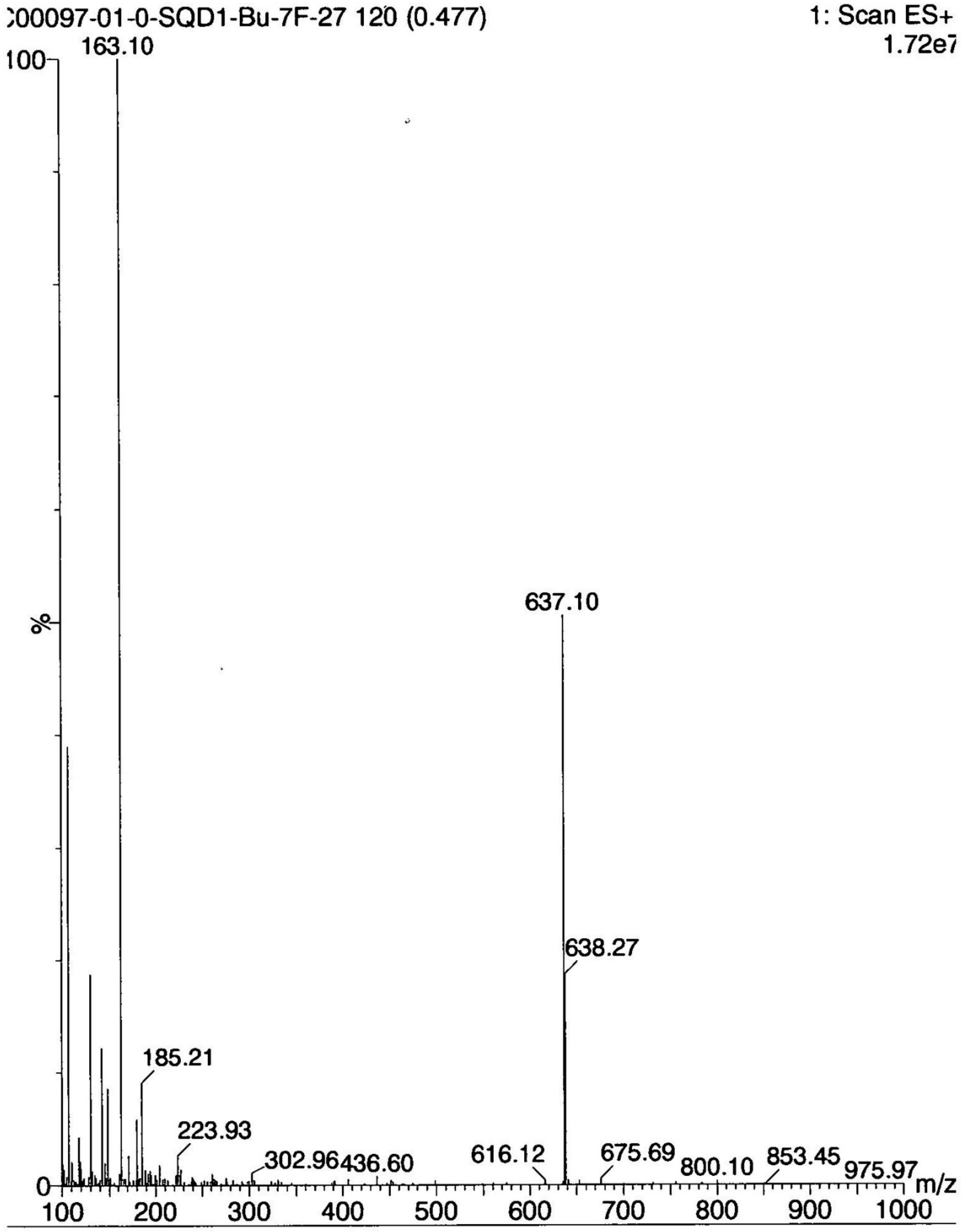

Figure S1. ESIMS (positive ion mode) spectrum of compound **1**.

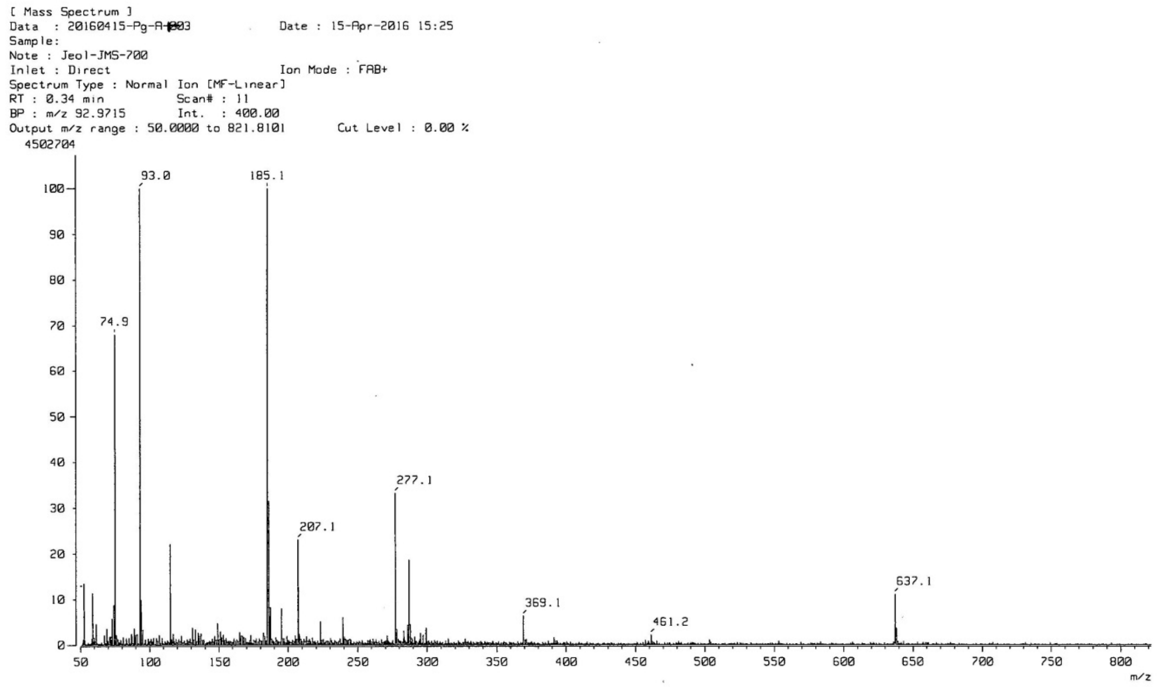

Figure S2. FABMS (positive ion mode) spectrum of compound **1**.

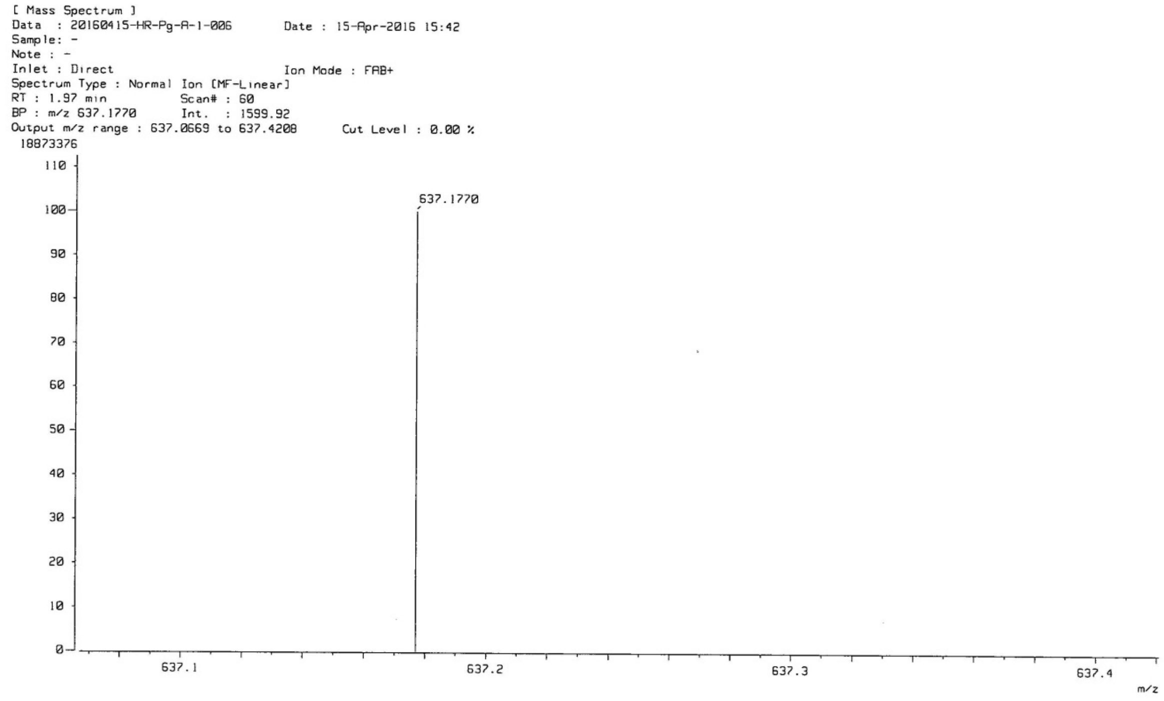

Figure S3. HRFABMS (positive ion mode) spectrum of compound **1**.

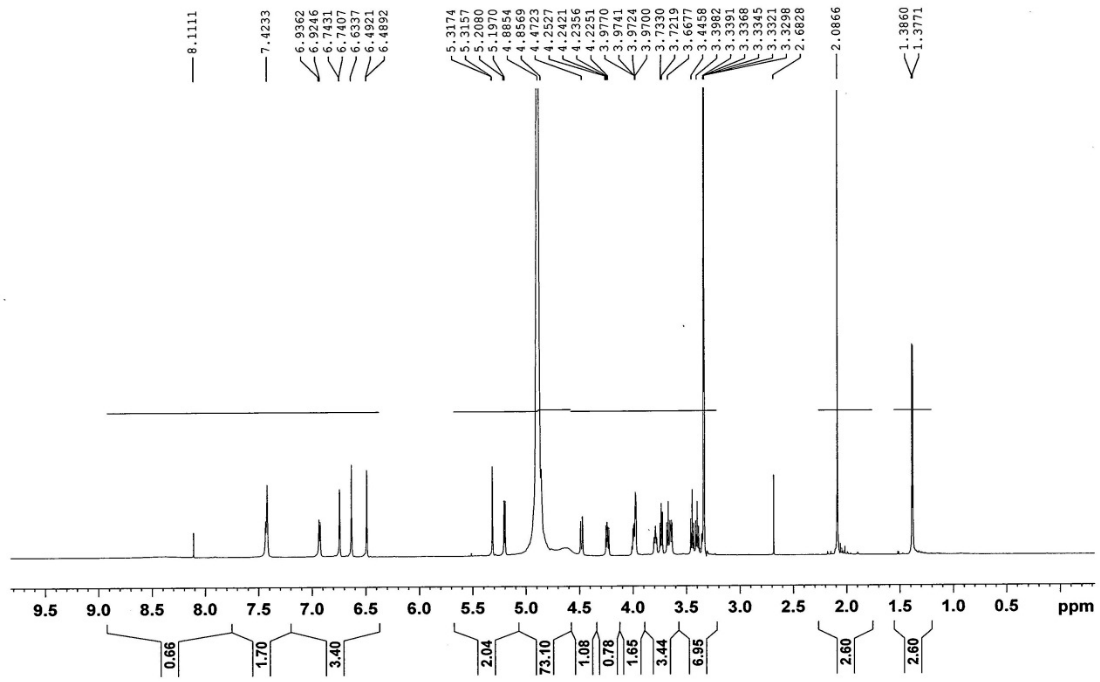

Figure S4. ^{1}H NMR spectrum of compound **1** (CD_3OD, 700 MHz).

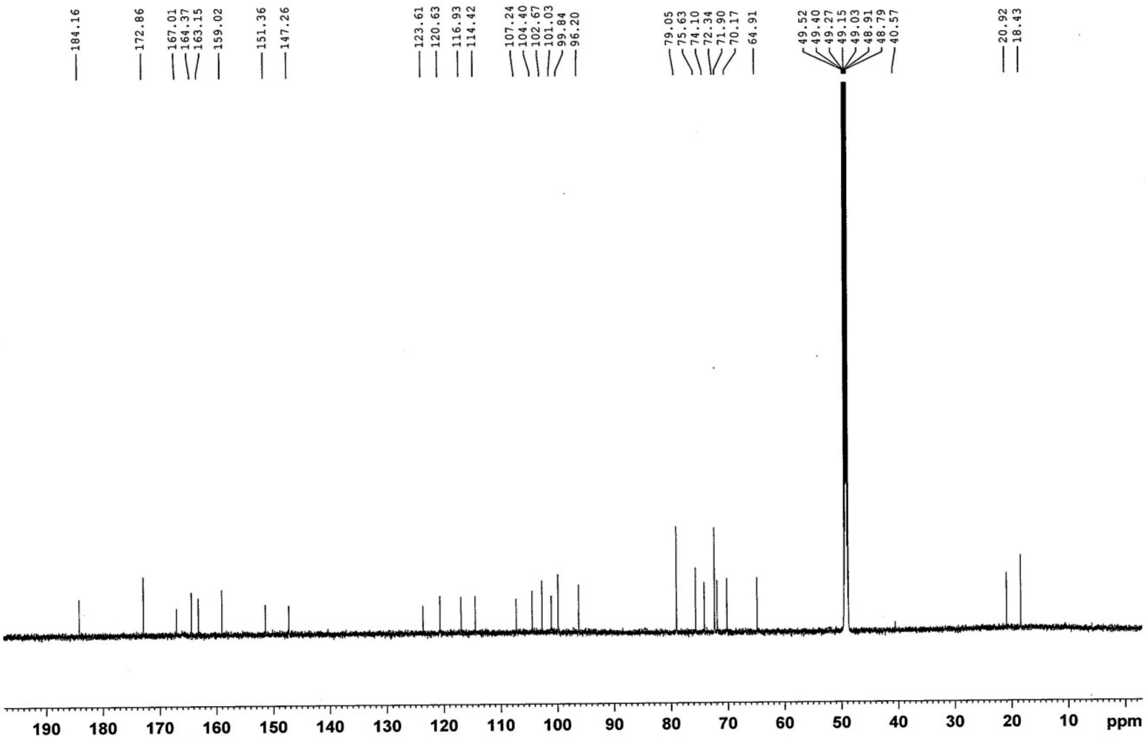

Figure S5. ^{13}C NMR spectrum of compound **1** (CD_3OD, 176 MHz).

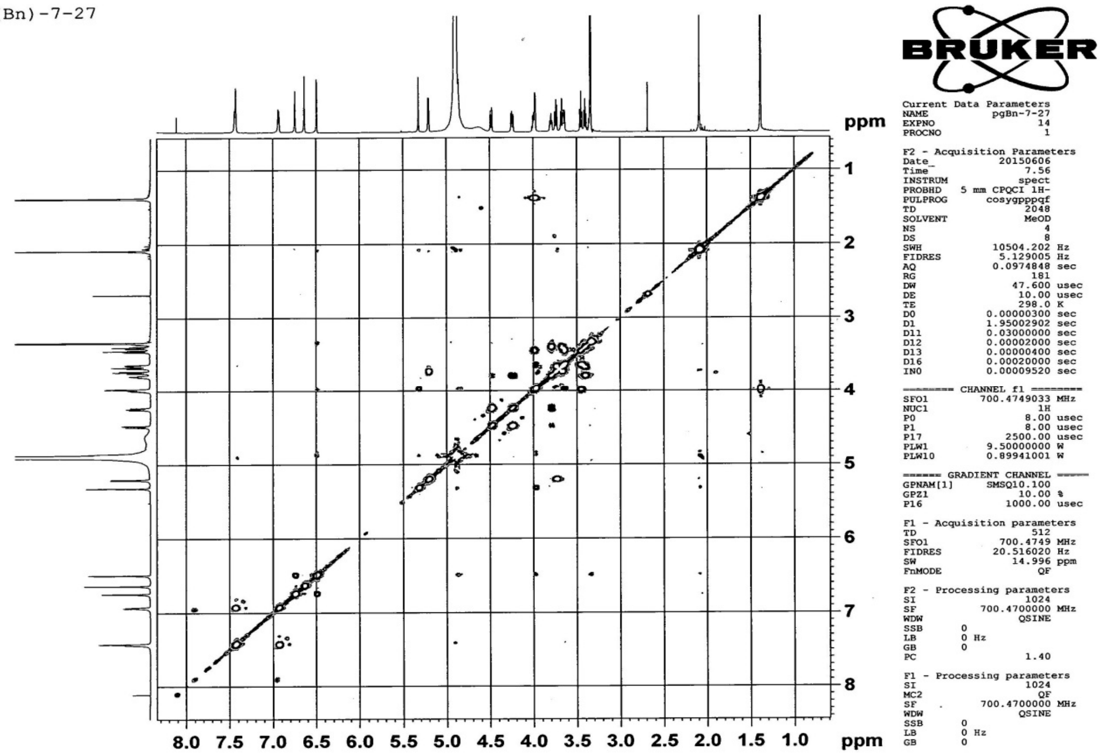

Figure S6. ^{1}H-^{1}H COSY spectrum of compound **1** (CD_3OD, 700 MHz).

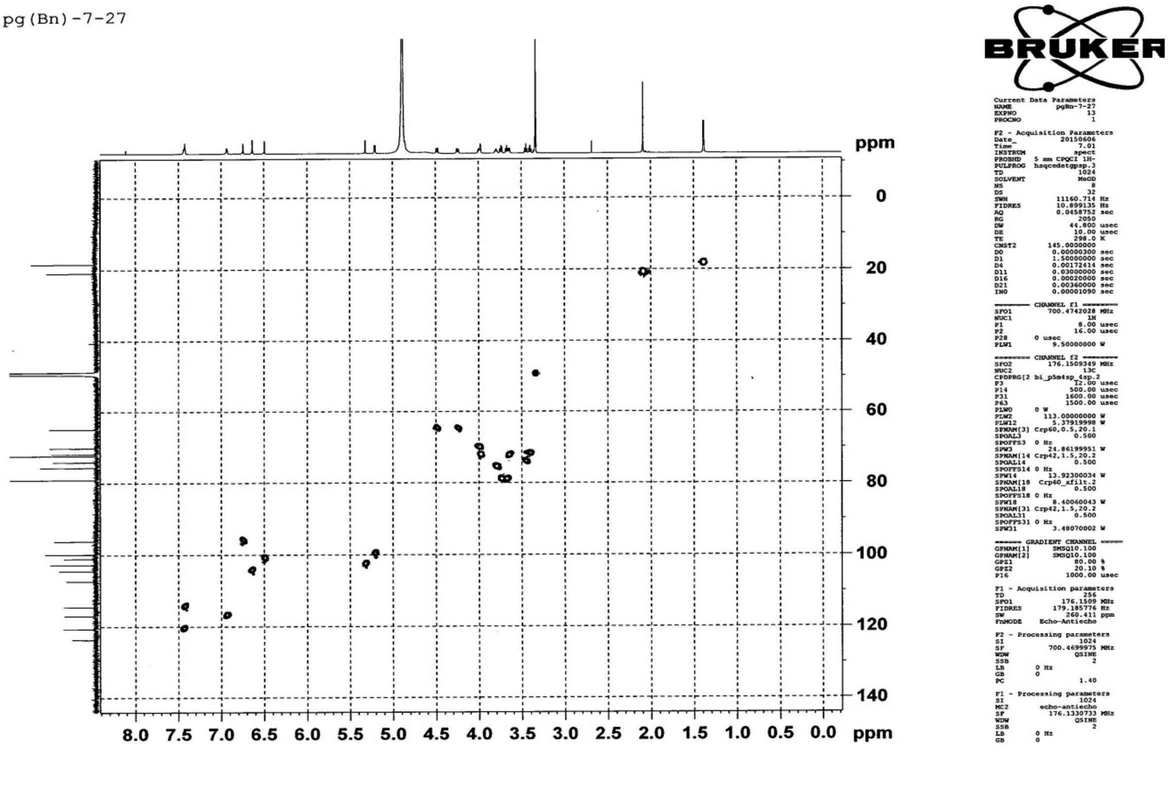

Figure S7. HSQC spectrum of compound **1** (CD_3OD, 700 MHz).

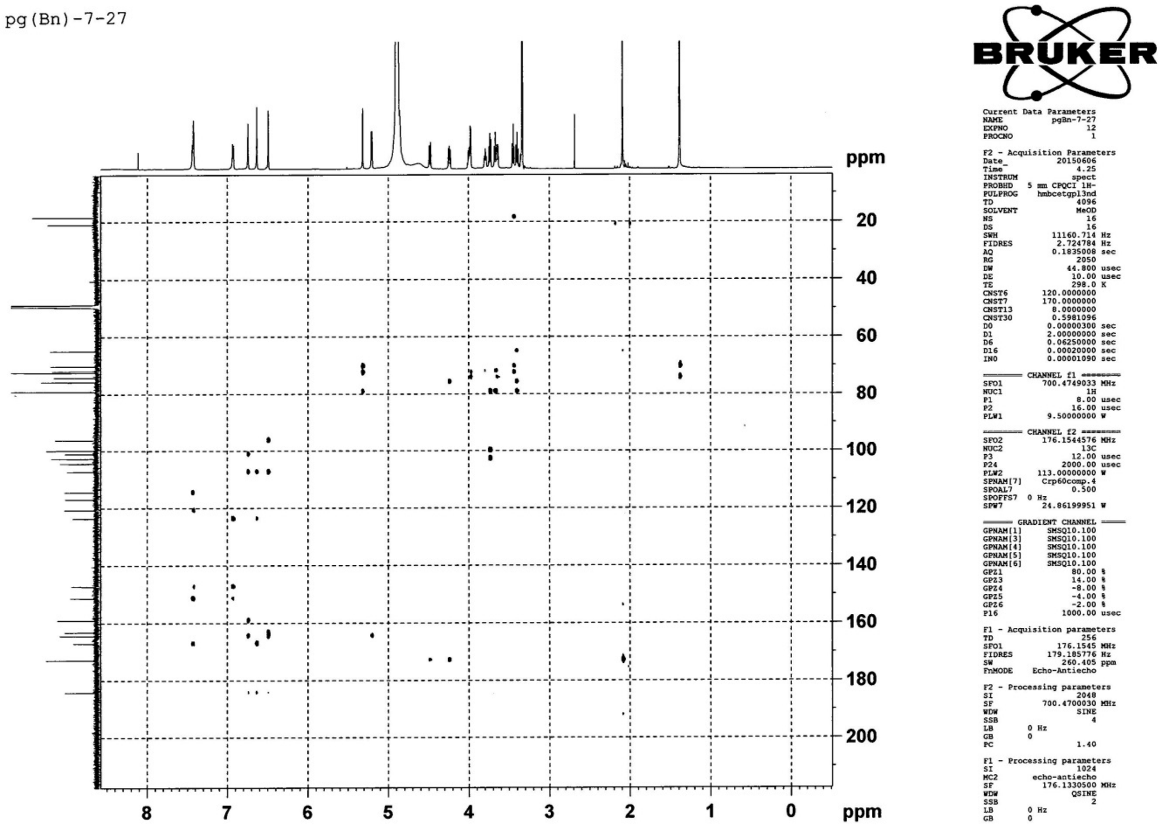

Figure S8. HMBC spectrum of compound **1** (CD_3OD, 700 MHz).

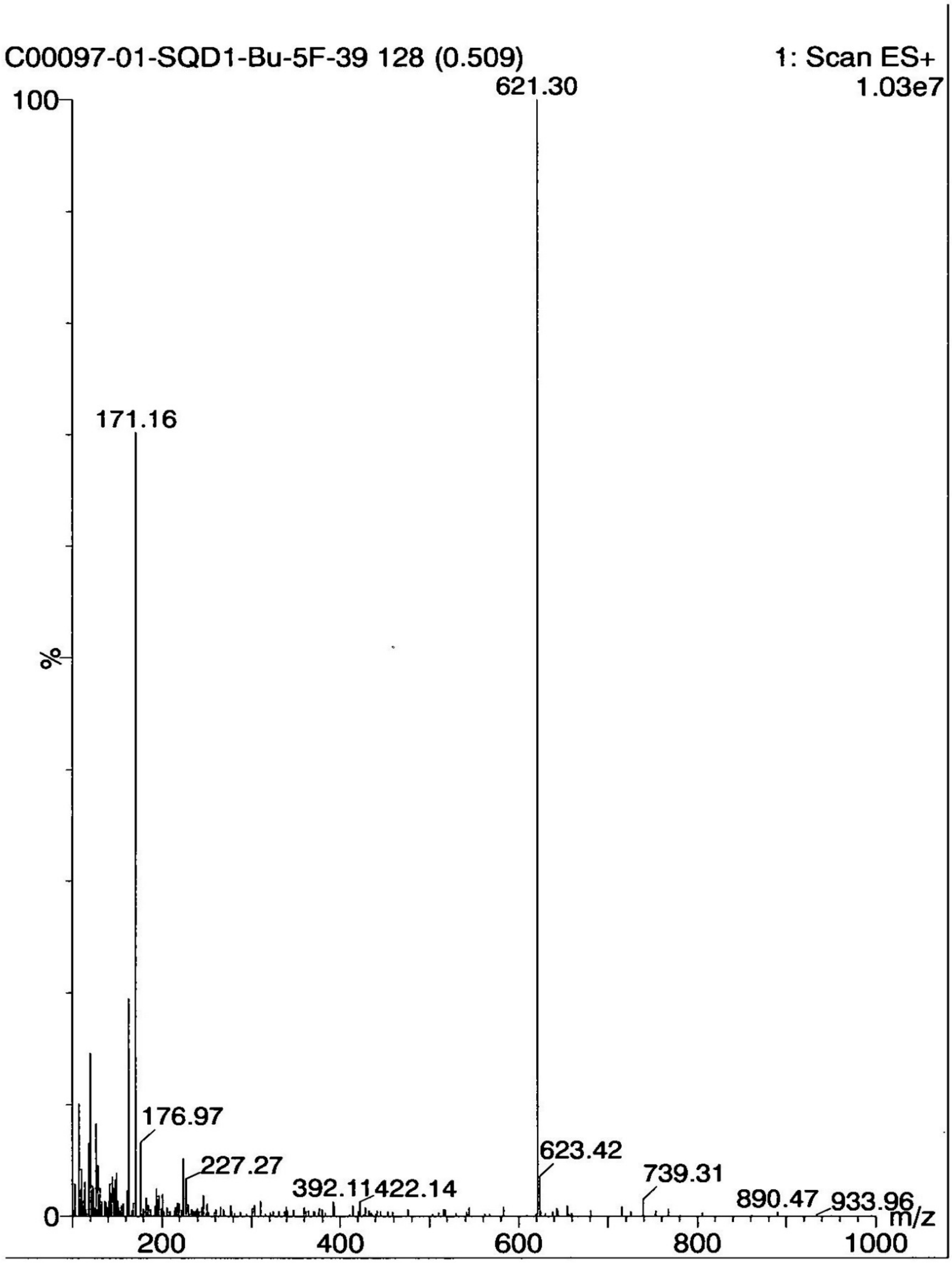

Figure S9. ESIMS (positive ion mode) spectrum of compound **2**.

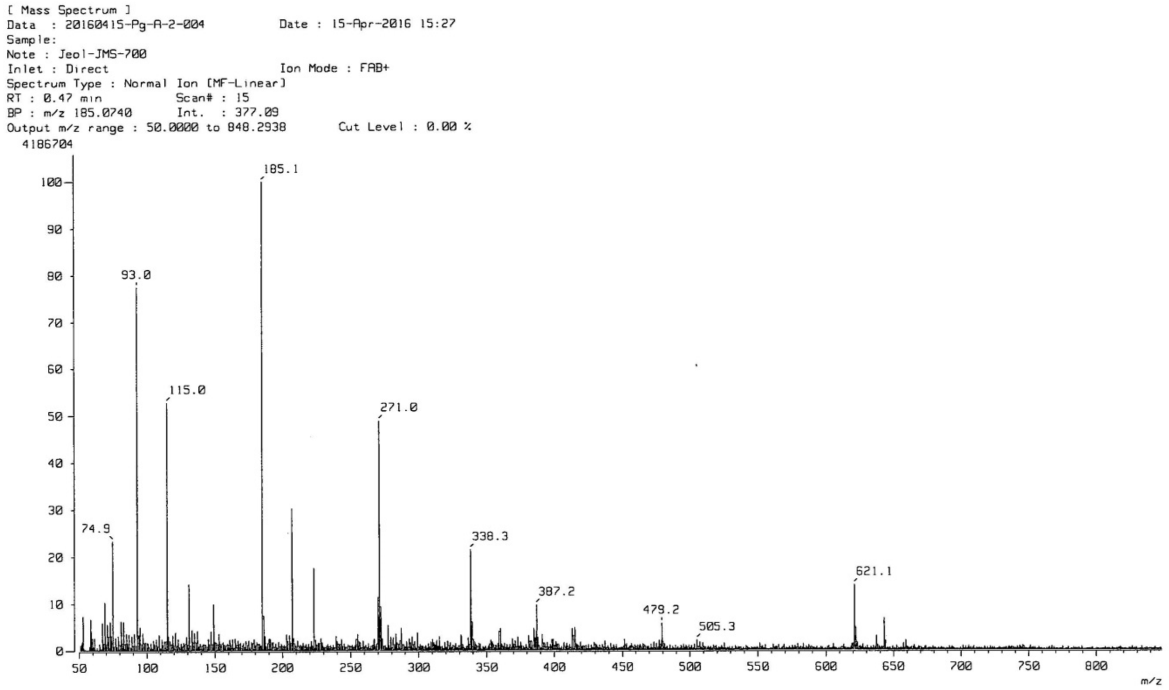

Figure S10. FABMS (positive ion mode) spectrum of compound **2**.

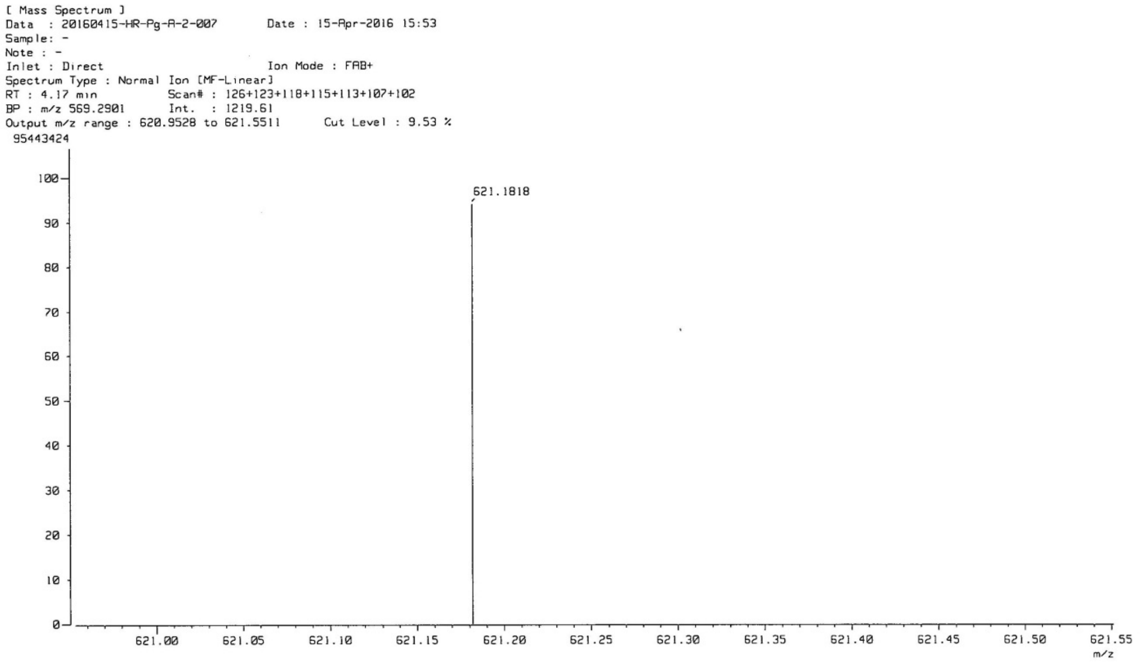

Figure S11. HRFABMS (positive ion mode) spectrum of compound **2**.

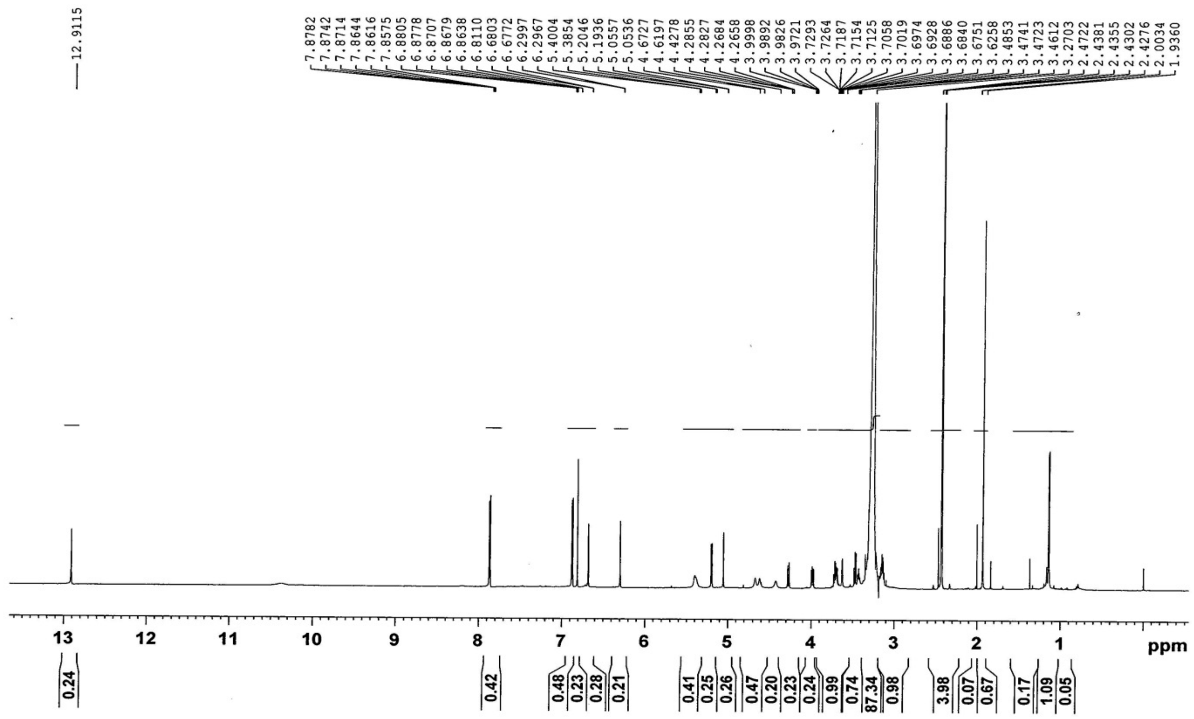

Figure S12. ^{1}H NMR spectrum of compound **2** (DMSO-d_6, 700 MHz).

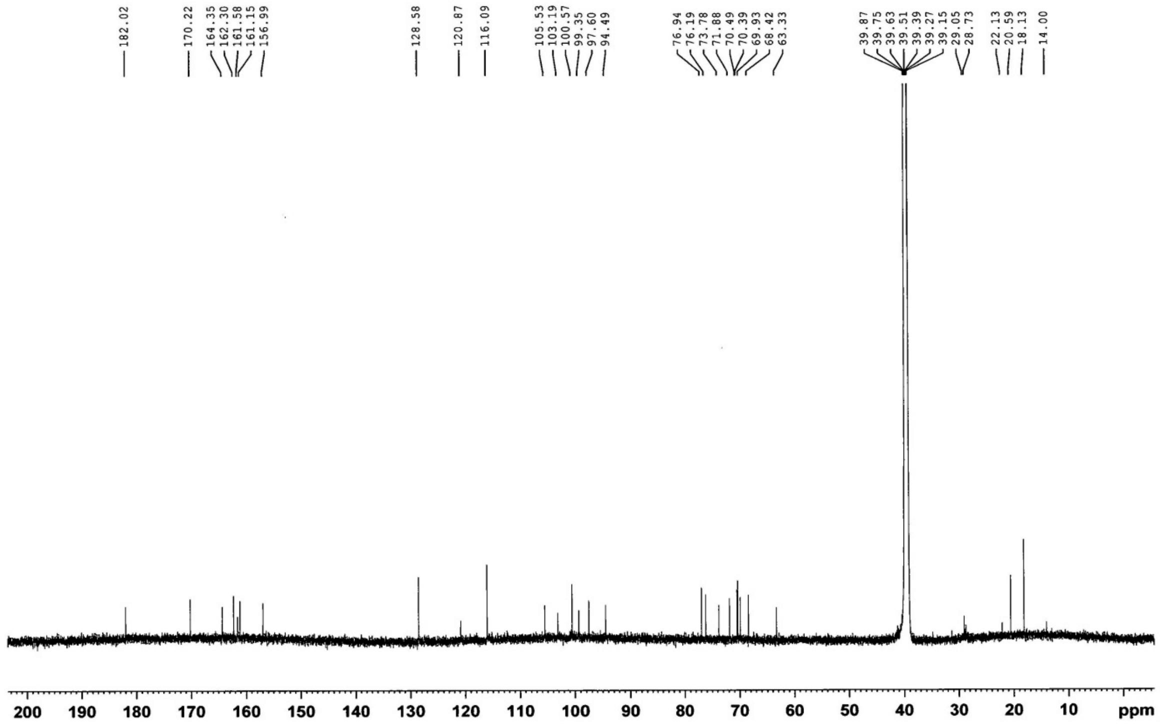

Figure S13. ^{13}C NMR spectrum of compound **2** (DMSO-d_6, 176 MHz).

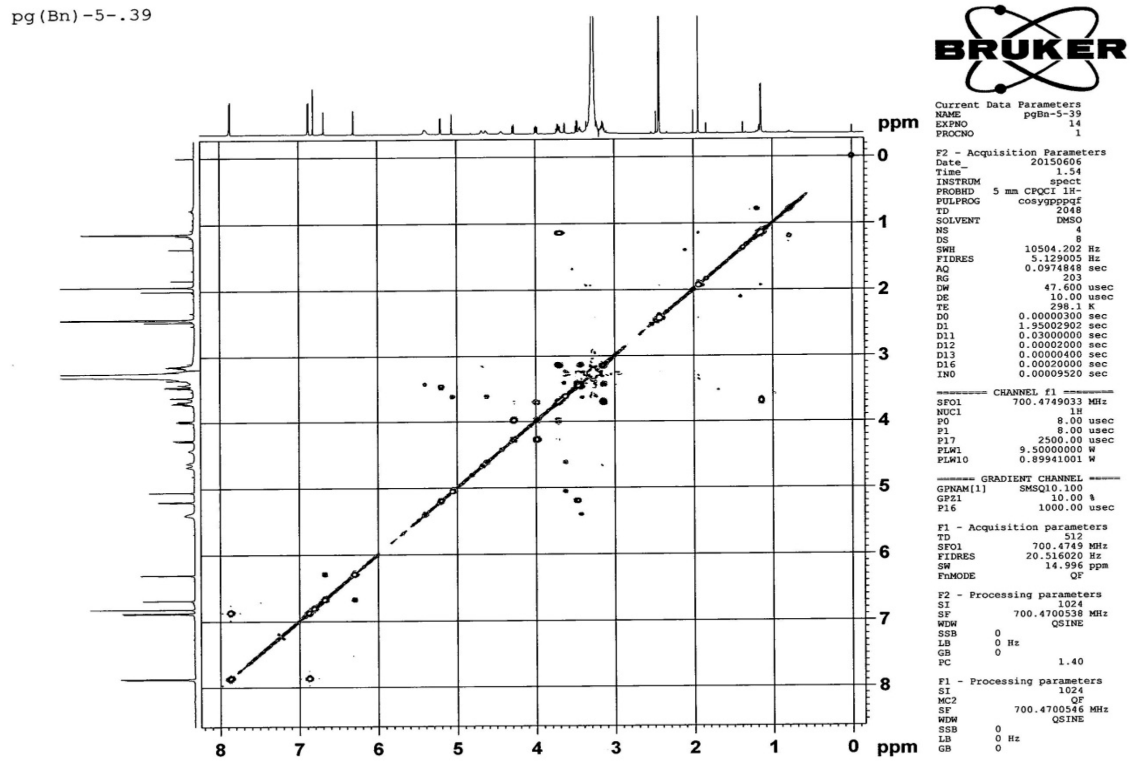

Figure S14. ^{1}H-^{1}H COSY spectrum of compound **2** (DMSO-*d*$_6$, 700 MHz).

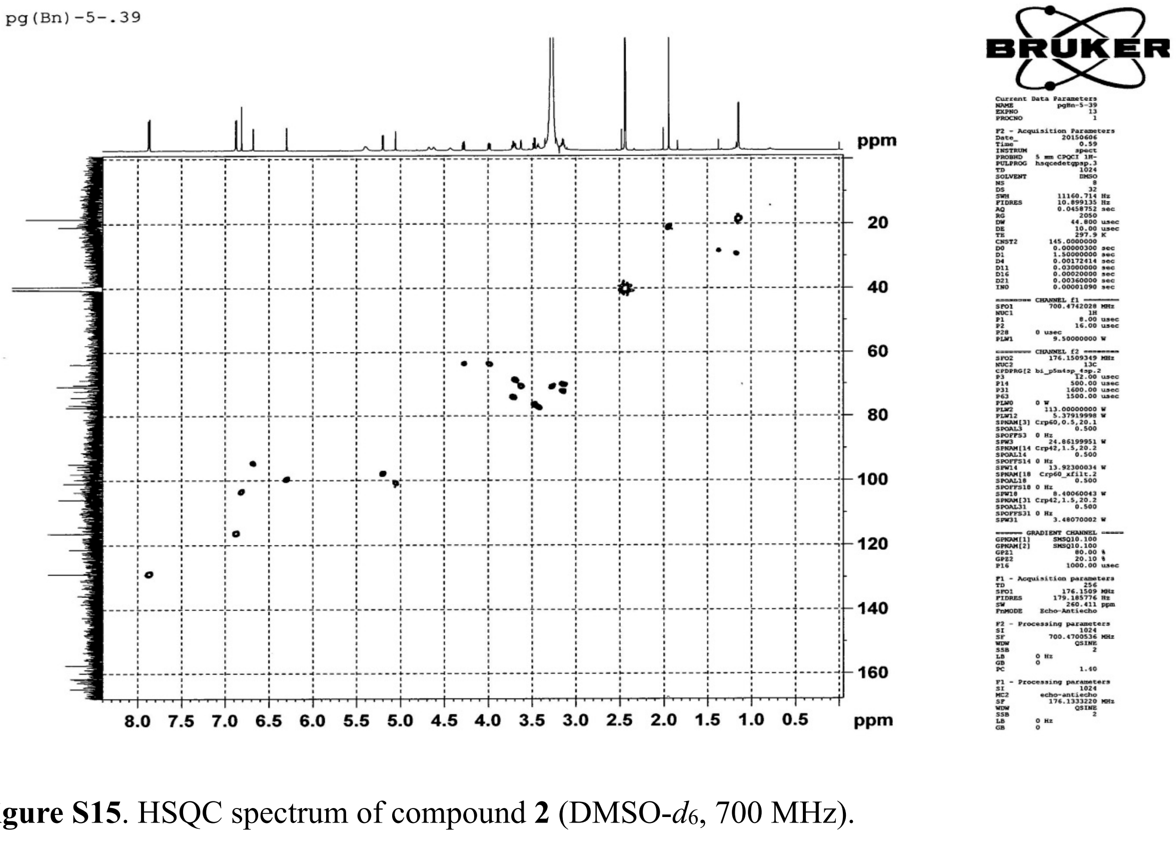

Figure S15. HSQC spectrum of compound **2** (DMSO-*d*$_6$, 700 MHz).

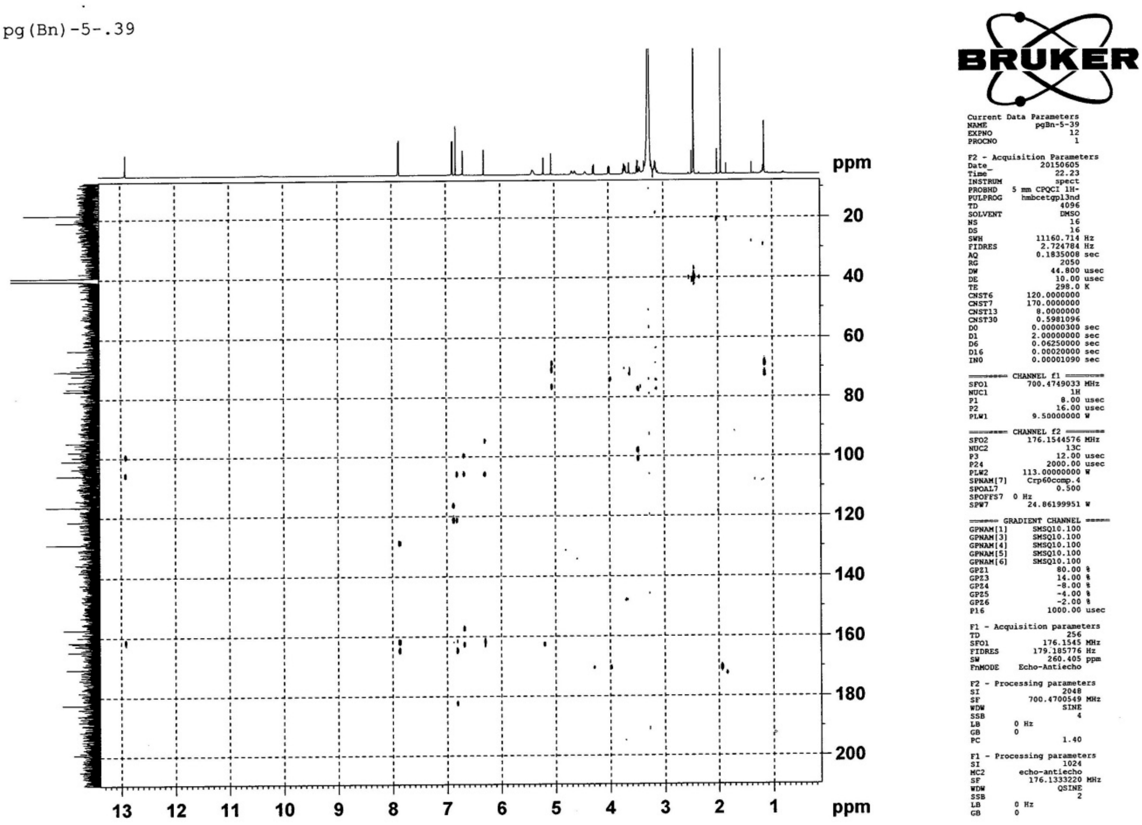

Figure S16. HMBC spectrum of compound **2** (DMSO-d_6, 700 MHz).
