# Supplementary material for: Ameliorative Effects of Flavonoids from Platycodon grandiflorus Aerial Parts on Alloxan-Induced Pancreatic Islet Damage in Zebrafish
Source: Nutrients. 2023 Apr 6;15(7):1798. doi: 10.3390/nu15071798 (PMC10096680; doi:10.3390/nu15071798)
Supplement: Supplementary file 1 [file nutrients-15-01798-s001.zip › nutrients-supporting information (Platycodon).docx]

**SUPPORTING INFORMATION**

**Ameliorative Effects of Flavonoids from *Platycodon grandiflorus* Aerial Part on Alloxan-Induced Pancreatic Islet Damage in Zebrafish**

Youn Hee Nam ^1,†^, Eun Bin Kim ^2,†^, Ji Eun Kang ^2^, Ju Su Kim ^2^, Yukyoung Jeon ^2^, Sung Woo Shin ^1^, Tong Ho Kang ^1, *^, and Jong Hwan Kwak ^2,*^

^1^ Department of Oriental Medicine Biotechnology, College of Life Sciences and Graduate School of Biotechnology, Kyung Hee University, Global Campus, Gyeonggi 17104, Korea; 01030084217@hanmail.net (Y.H.N.); 01073205620@khu.ac.kr (S.W.S.)

^2^ School of Pharmacy, Sungkyunkwan University, Suwon, Gyeonggi-do 16419, Republic of Korea; eunbin.kim423@gmail.com (E.B.K.); rkdwlddms789@gmail.com (J.E.K.); cnc0315@daum.net (J.S.K.); jeon1112@skku.edu (Y.J.)

***** Correspondence: panjae@khu.ac.kr (T.H.K.); jhkwak@skku.edu (J.H.K.); Tel.: +82-31-201-3862 (T.H.K.); +82-31-290-7745(J.H.K.); Fax: +82-303-0300-0030 (T.H.K.); +82-31-292-8800 (J.H.K.)

^†^ These two authors contributed equally to this work.

**List of Contents**

**Figure S1**. ESIMS (positive ion mode) spectrum of compound **1**.

**Figure S2**. FABMS (positive ion mode) spectrum of compound **1**.

**Figure S3**. HRFABMS (positive ion mode) spectrum of compound **1**.

**Figure S4**. ^1^H NMR spectrum of compound **1** (CD_3_OD, 700 MHz).

**Figure S5**. ^13^C NMR spectrum of compound **1** (CD_3_OD, 176 MHz).

**Figure S6**. ^1^H-^1^H COSY spectrum of compound **1** (CD_3_OD, 700 MHz).

**Figure S7**. HSQC spectrum of compound **1** (CD_3_OD, 700 MHz).

**Figure S8**. HMBC spectrum of compound **1** (CD_3_OD, 700 MHz).

**Figure S9**. ESIMS (positive ion mode) spectrum of compound **2**.

**Figure S10**. FABMS (positive ion mode) spectrum of compound **2**.

**Figure S11**. HRFABMS (positive ion mode) spectrum of compound **2**.

**Figure S12**. ^1^H NMR spectrum of compound **2** (DMSO-*d*_6_, 700 MHz).

**Figure S13**. ^13^C NMR spectrum of compound **2** (DMSO-*d*_6_, 176 MHz).

**Figure S14**. ^1^H-^1^H COSY spectrum of compound **2** (DMSO-*d*_6_, 700 MHz).

**Figure S15**. HSQC spectrum of compound **2** (DMSO-*d*_6_, 700 MHz).

**Figure S16**. HMBC spectrum of compound **2** (DMSO-*d*_6_, 700 MHz).


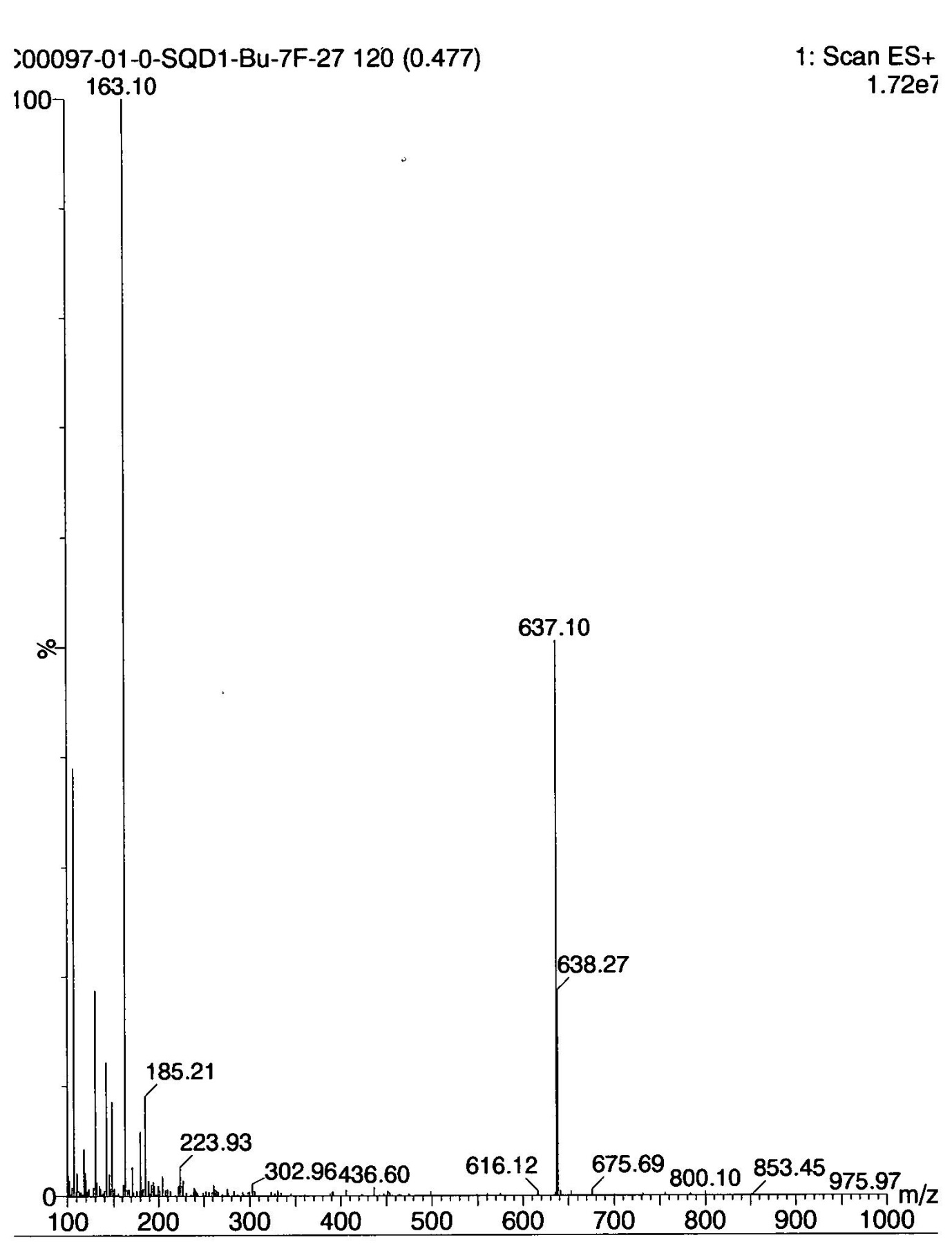


**Figure S1**. ESIMS (positive ion mode) spectrum of compound **1**.


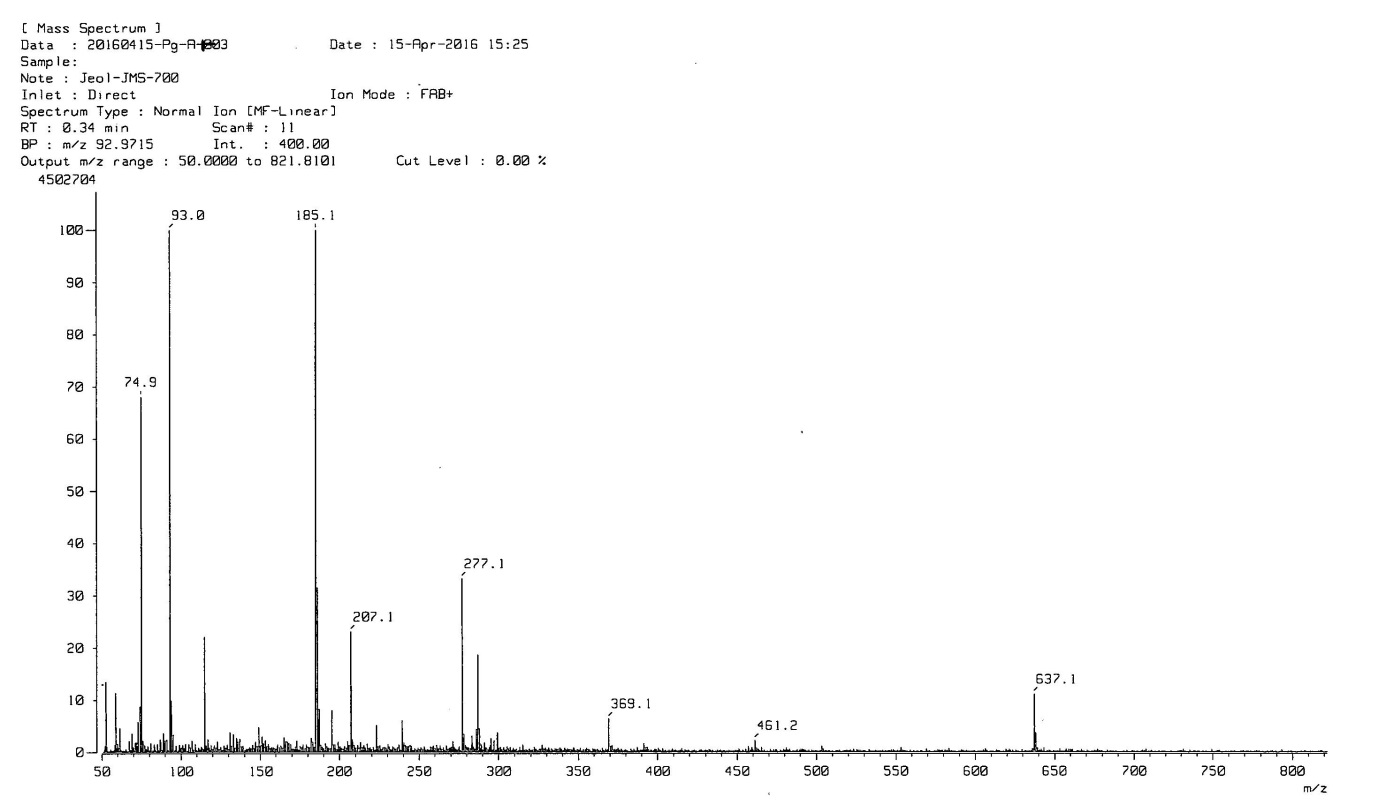


**Figure S2**. FABMS (positive ion mode) spectrum of compound **1**.


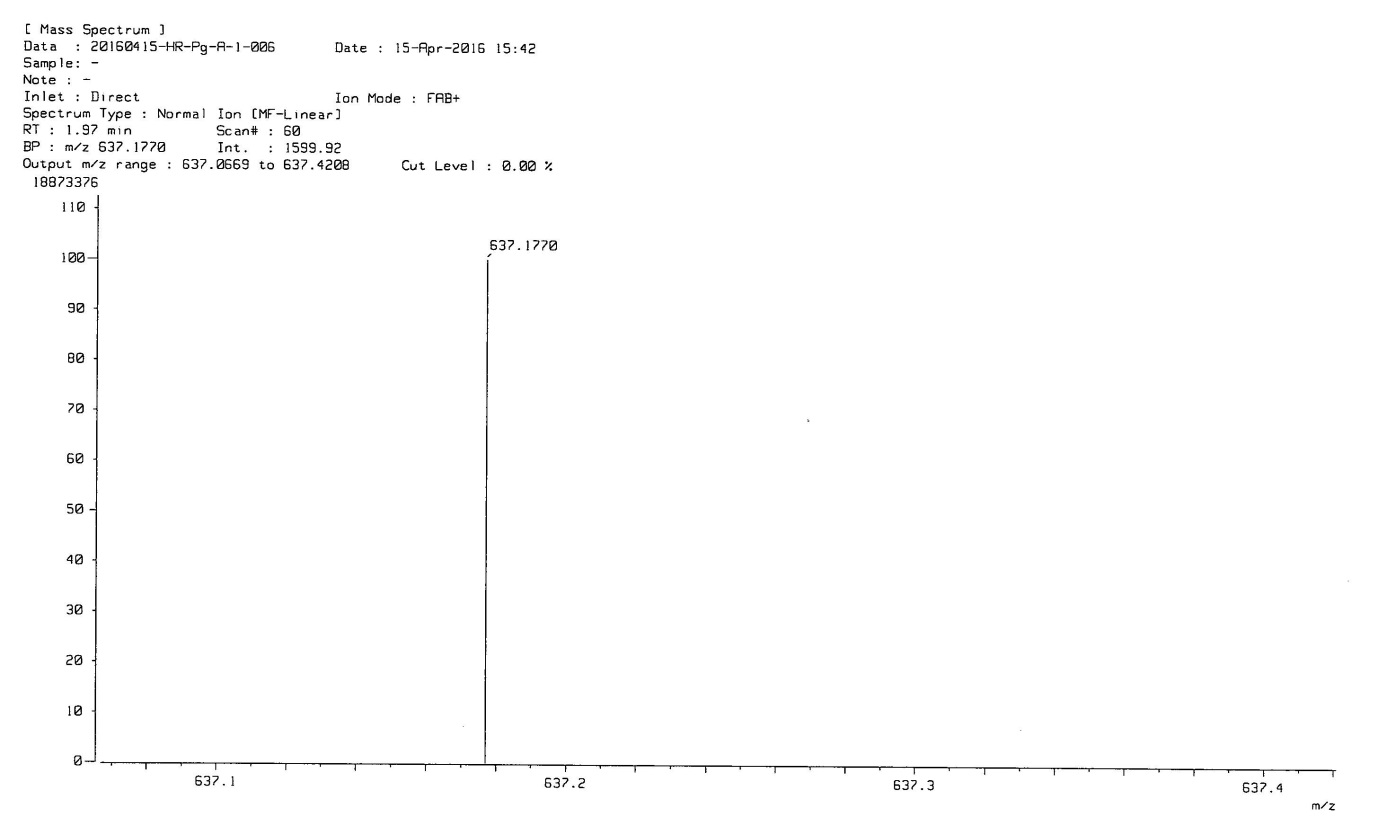


**Figure S3**. HRFABMS (positive ion mode) spectrum of compound **1**.

**
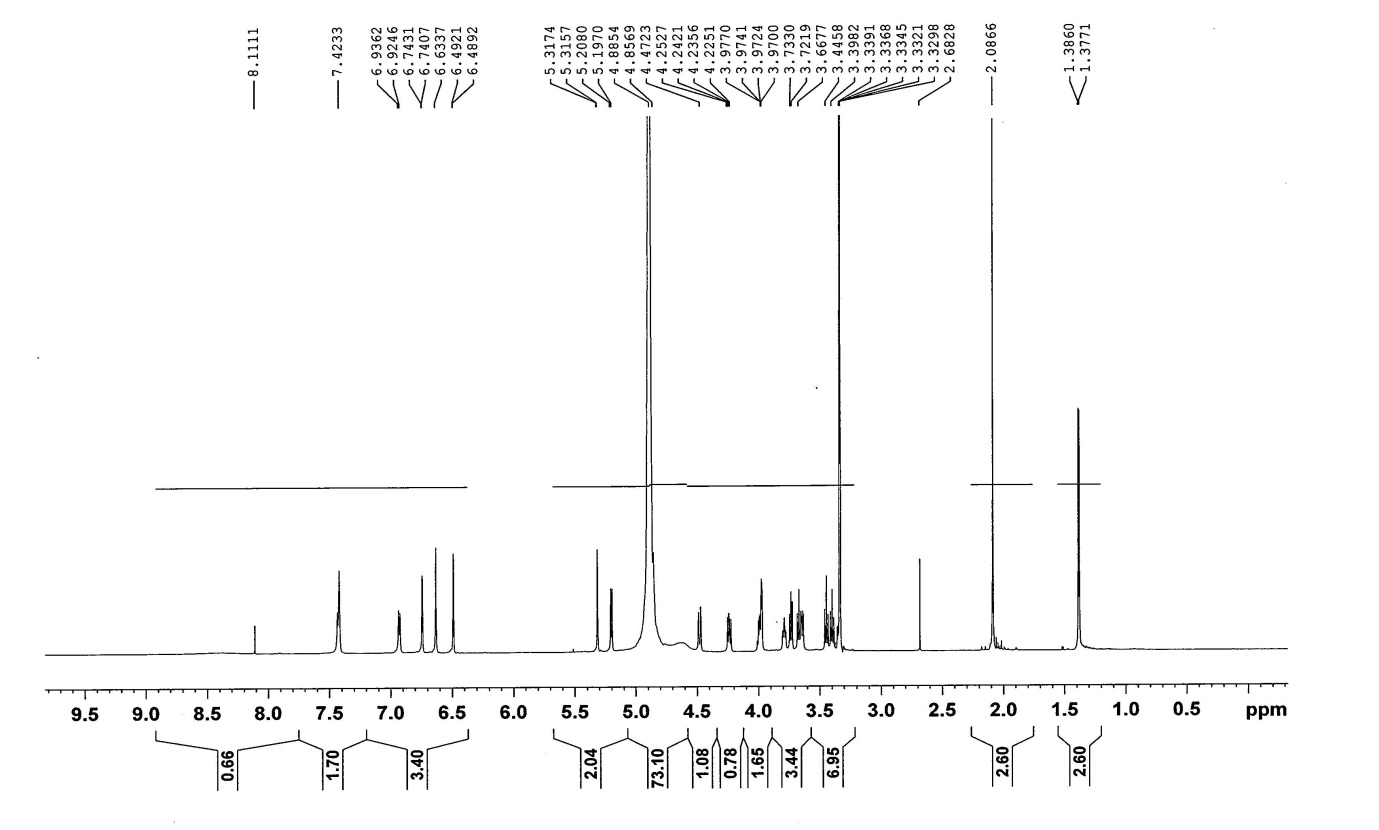
**

**Figure S4**. ^1^H NMR spectrum of compound **1** (CD_3_OD, 700 MHz).


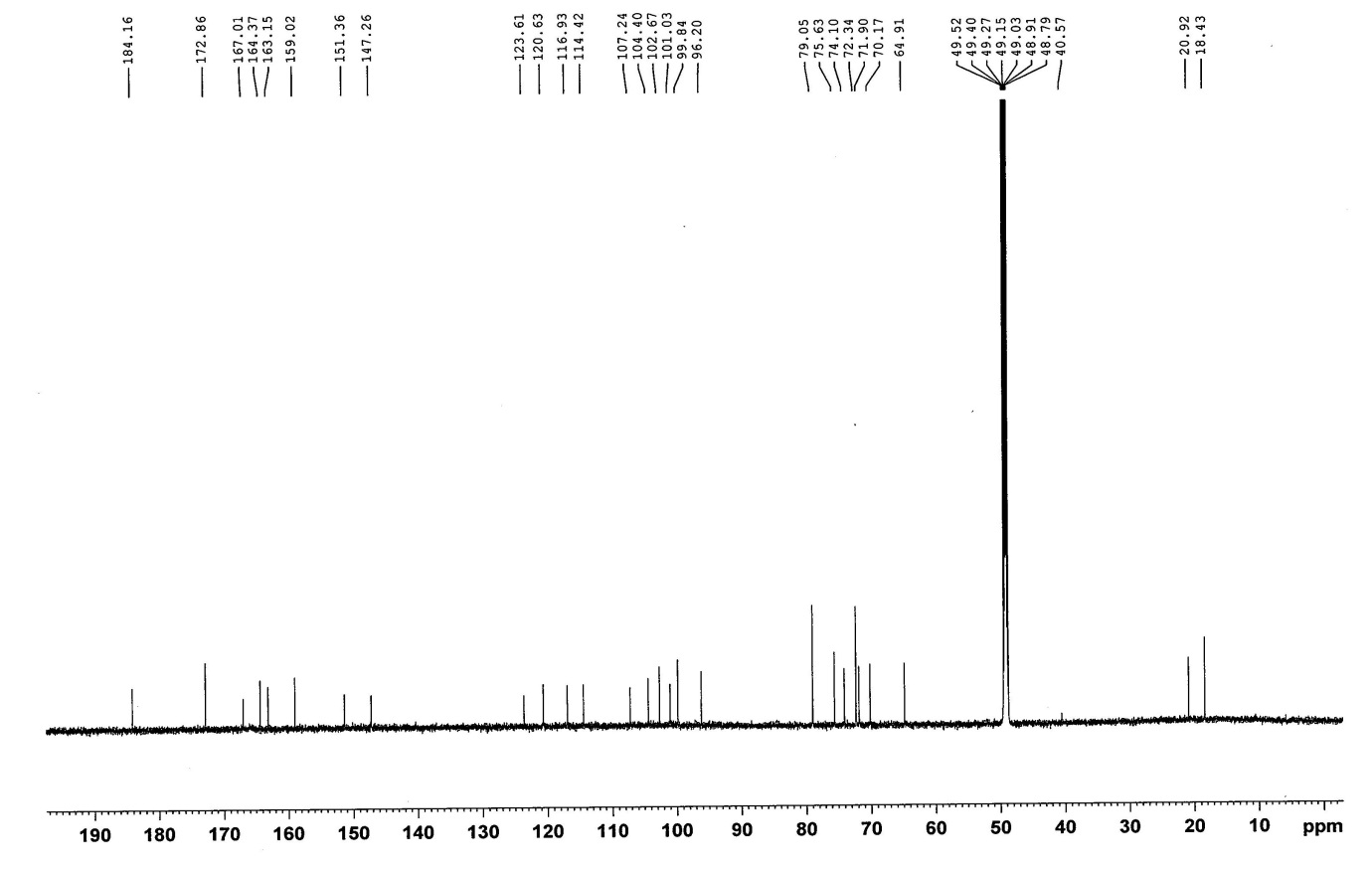


**Figure S5**. ^13^C NMR spectrum of compound **1** (CD_3_OD, 176 MHz).

**
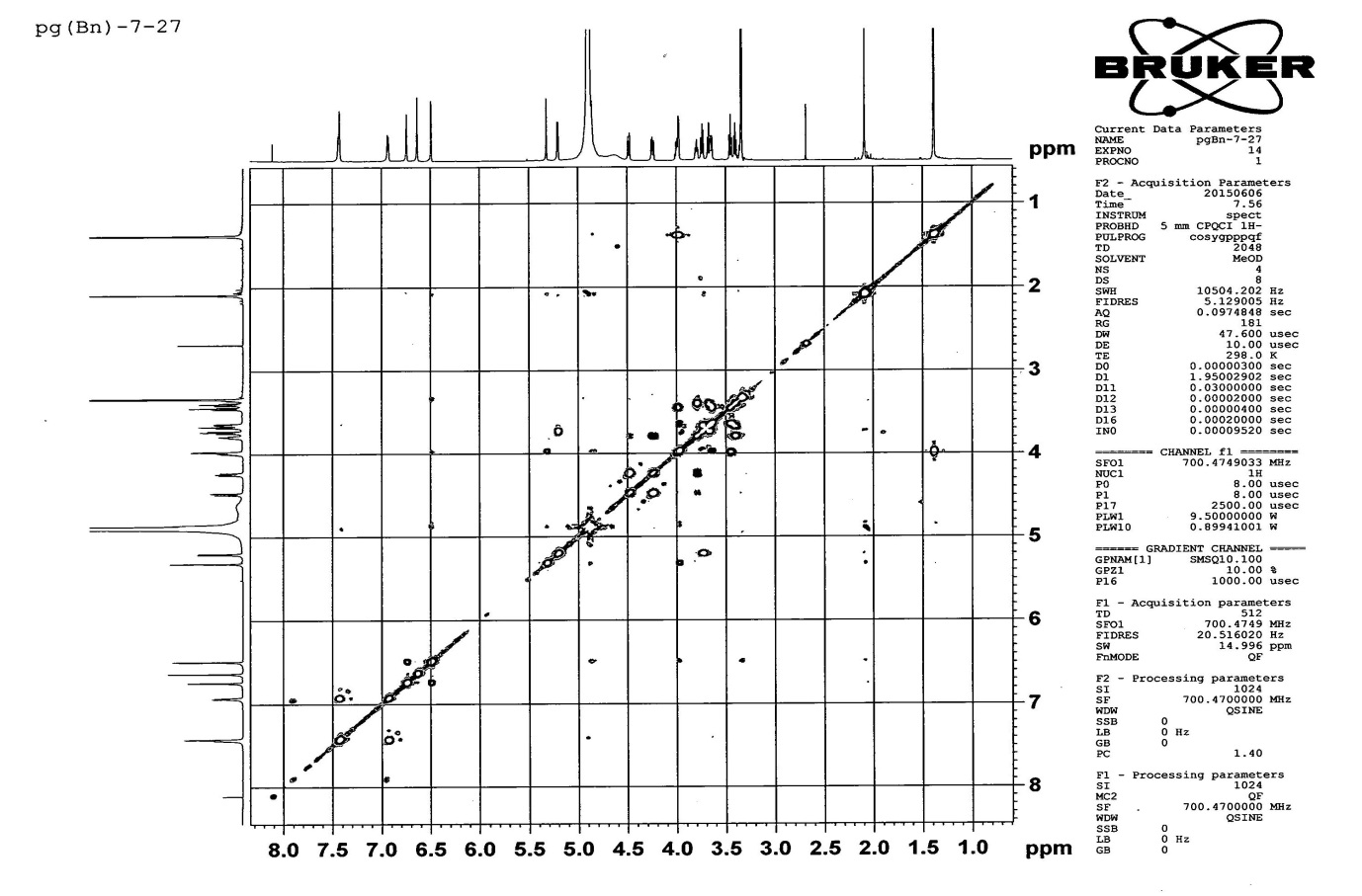
**

**Figure S6**. ^1^H-^1^H COSY spectrum of compound **1** (CD_3_OD, 700 MHz).


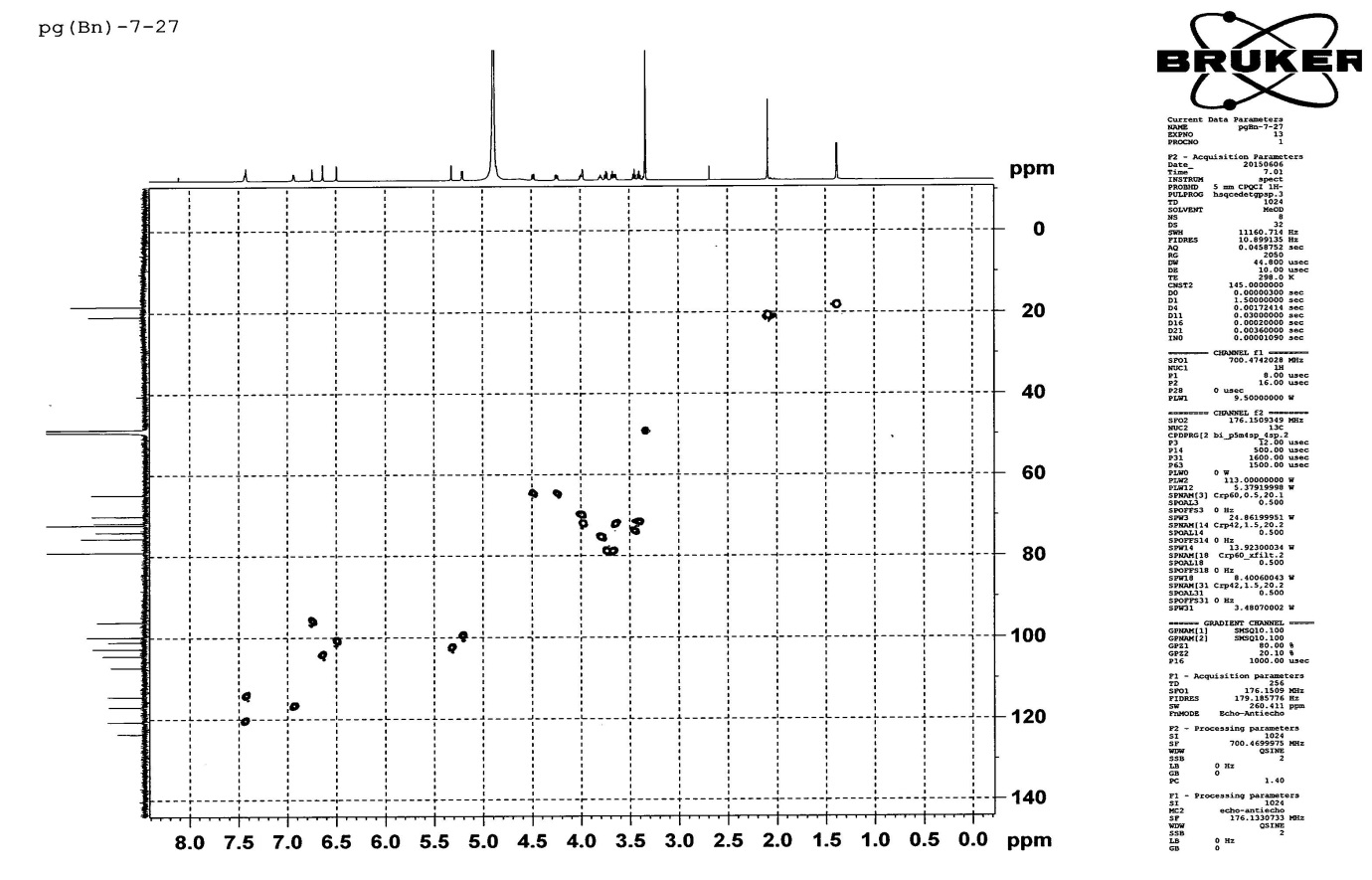


**Figure S7**. HSQC spectrum of compound **1** (CD_3_OD, 700 MHz).


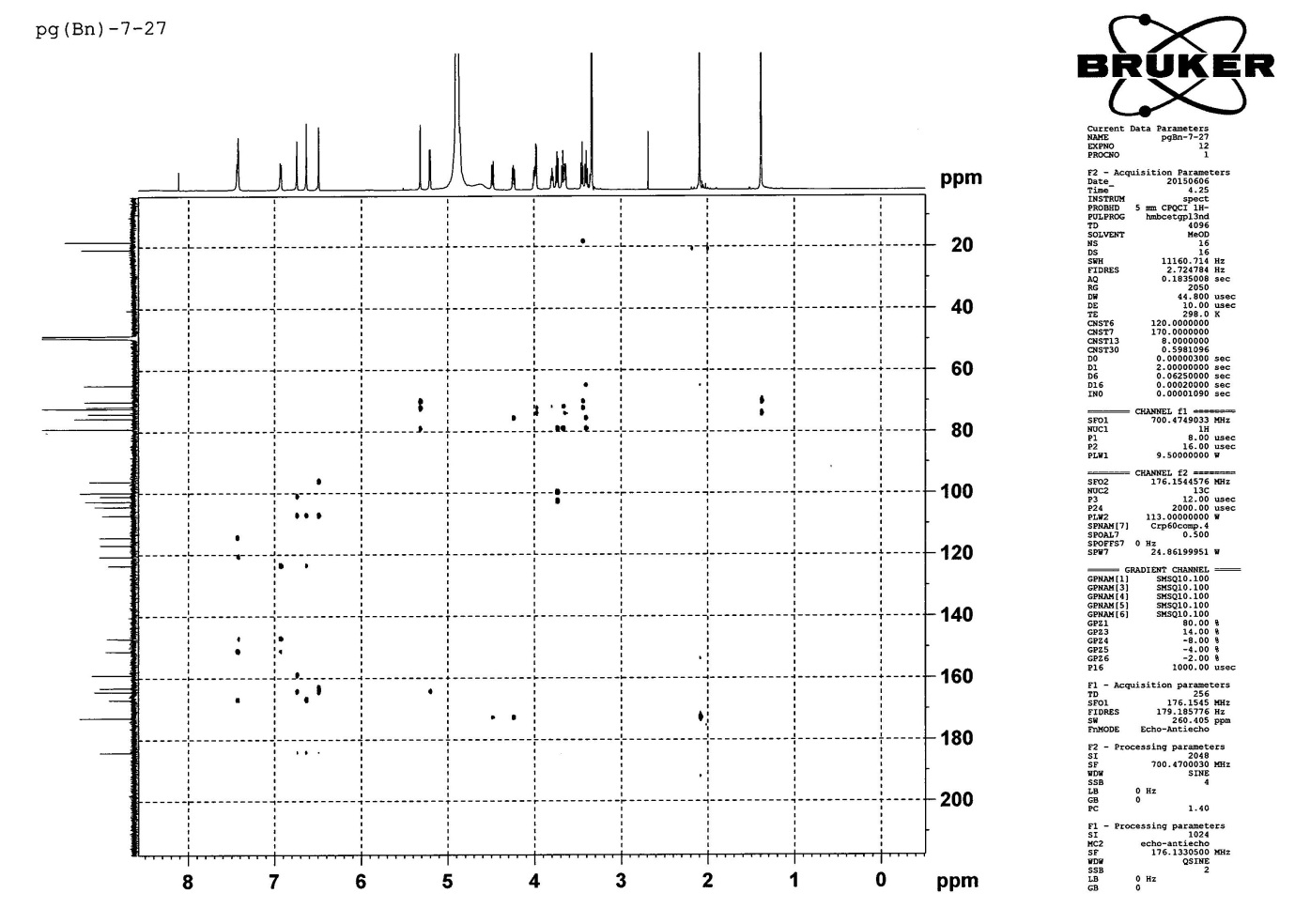


**Figure S8**. HMBC spectrum of compound **1** (CD_3_OD, 700 MHz).


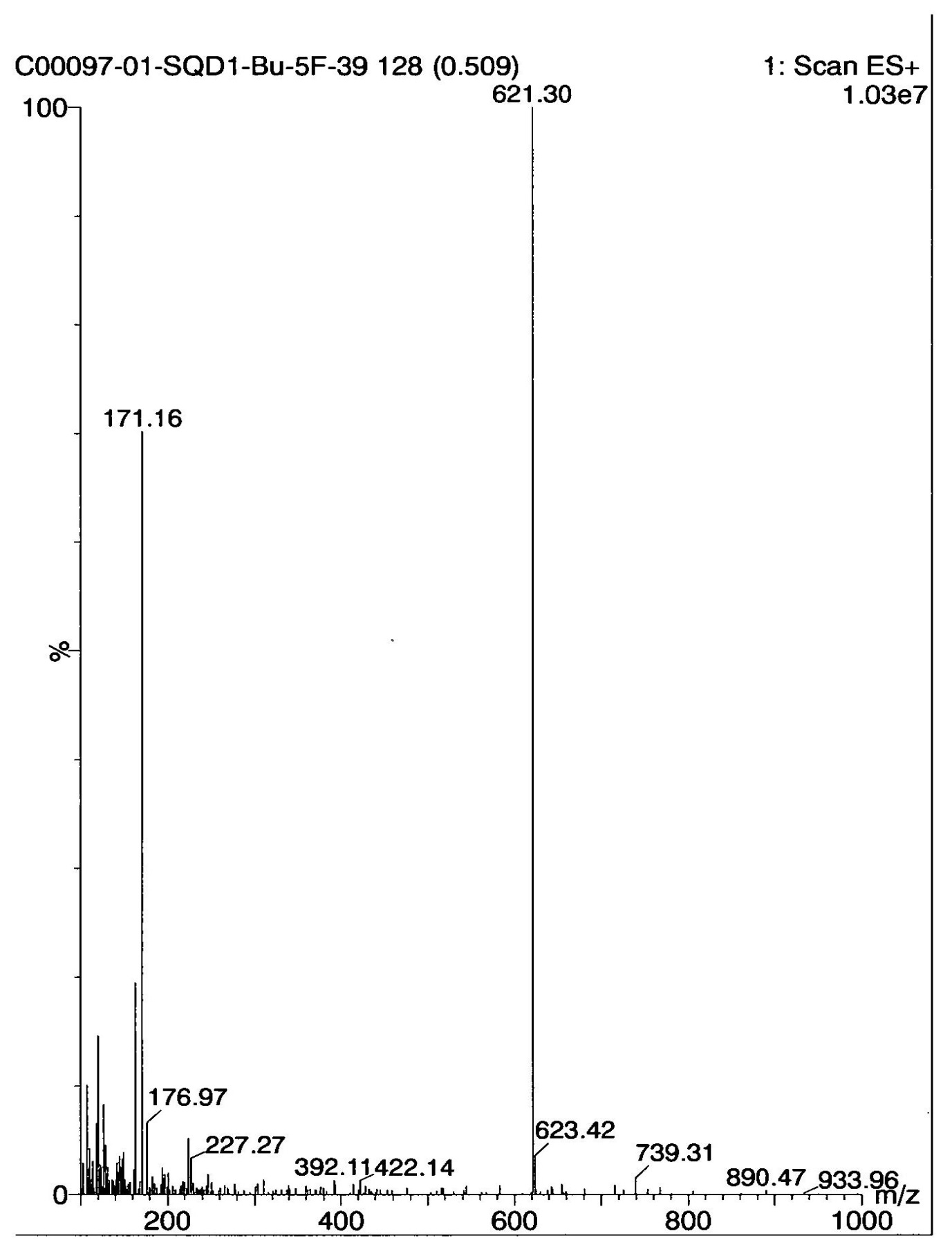


**Figure S9**. ESIMS (positive ion mode) spectrum of compound **2**.


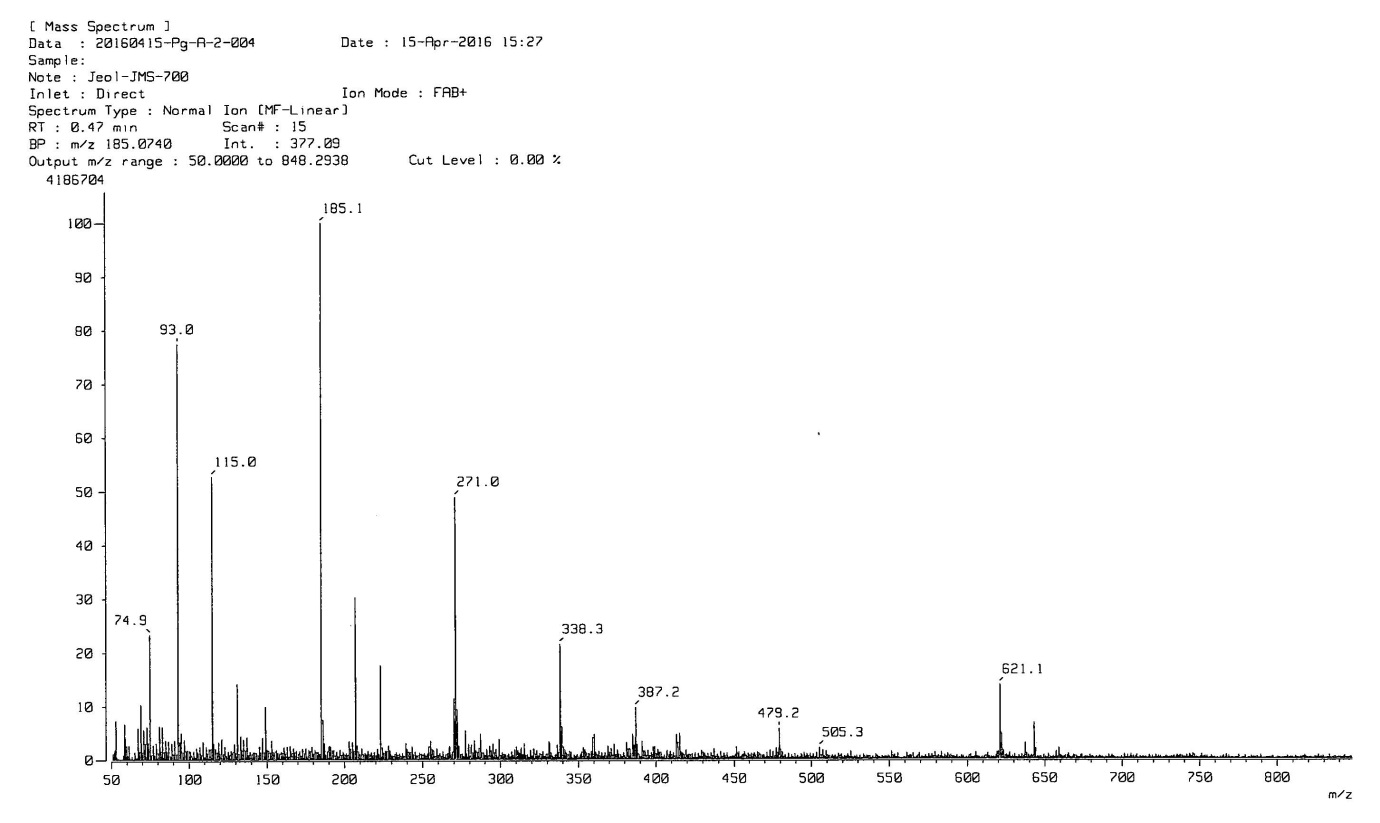


**Figure S10**. FABMS (positive ion mode) spectrum of compound **2**.


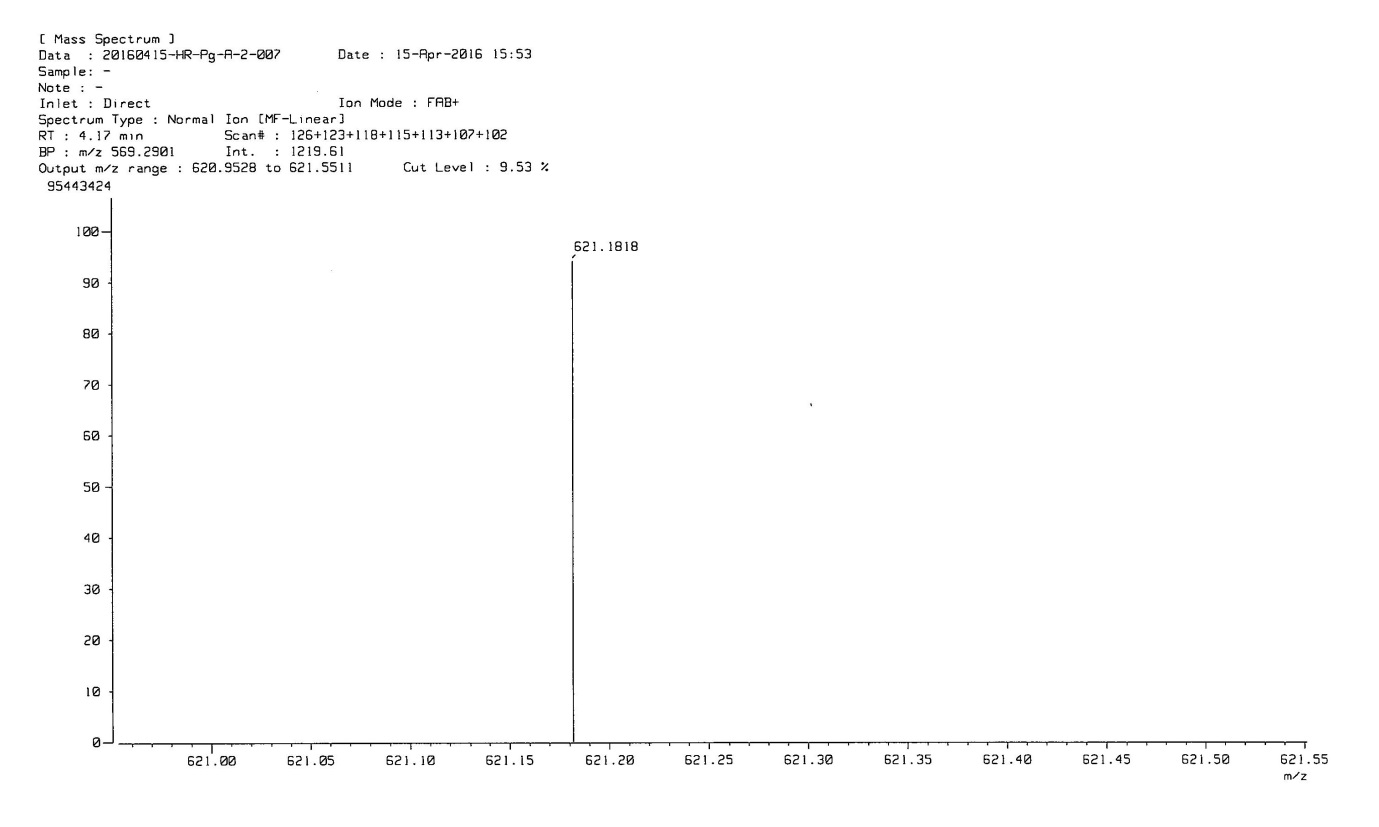


**Figure S11**. HRFABMS (positive ion mode) spectrum of compound **2**.


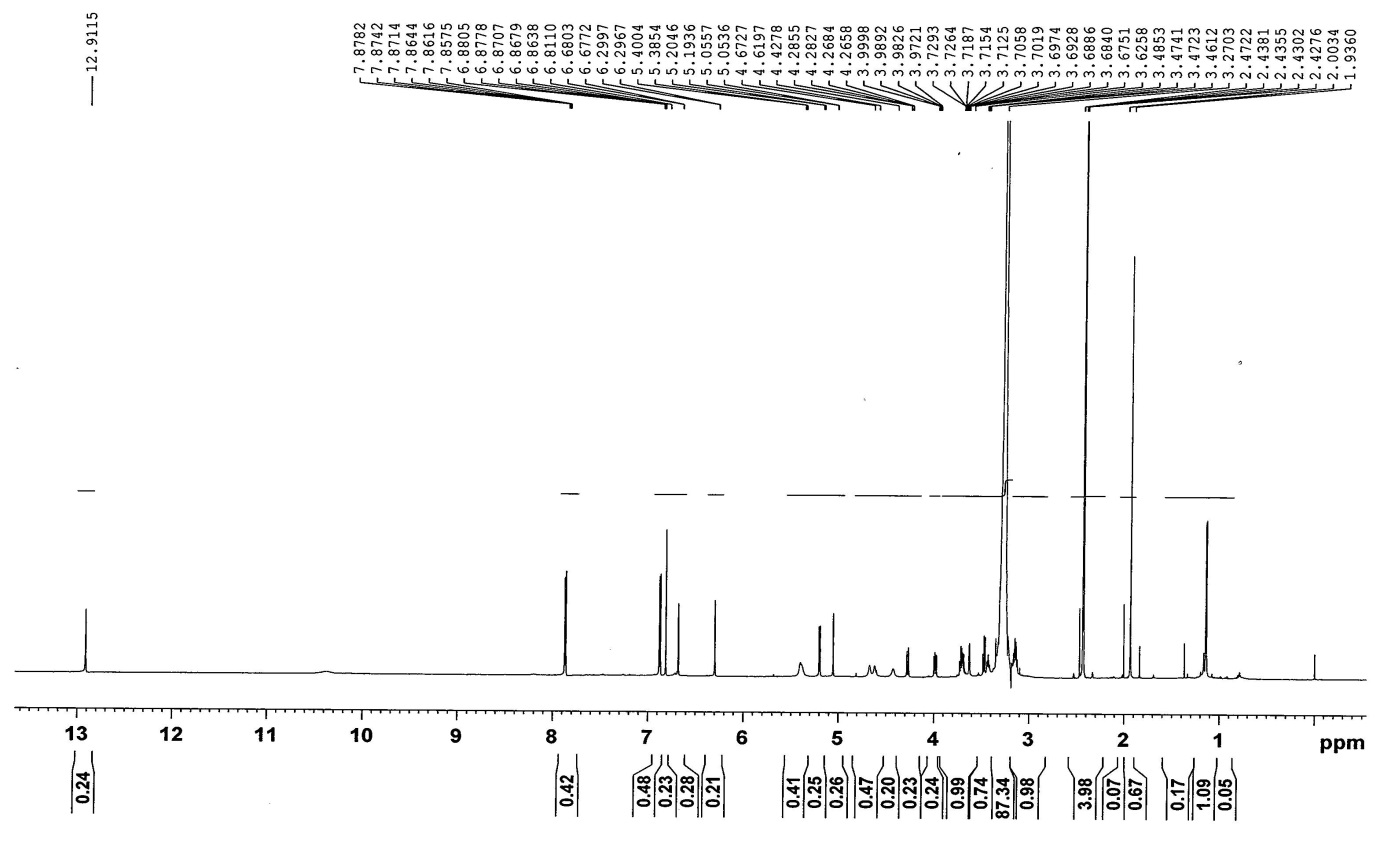


**Figure S12**. ^1^H NMR spectrum of compound **2** (DMSO-*d*_6_, 700 MHz).


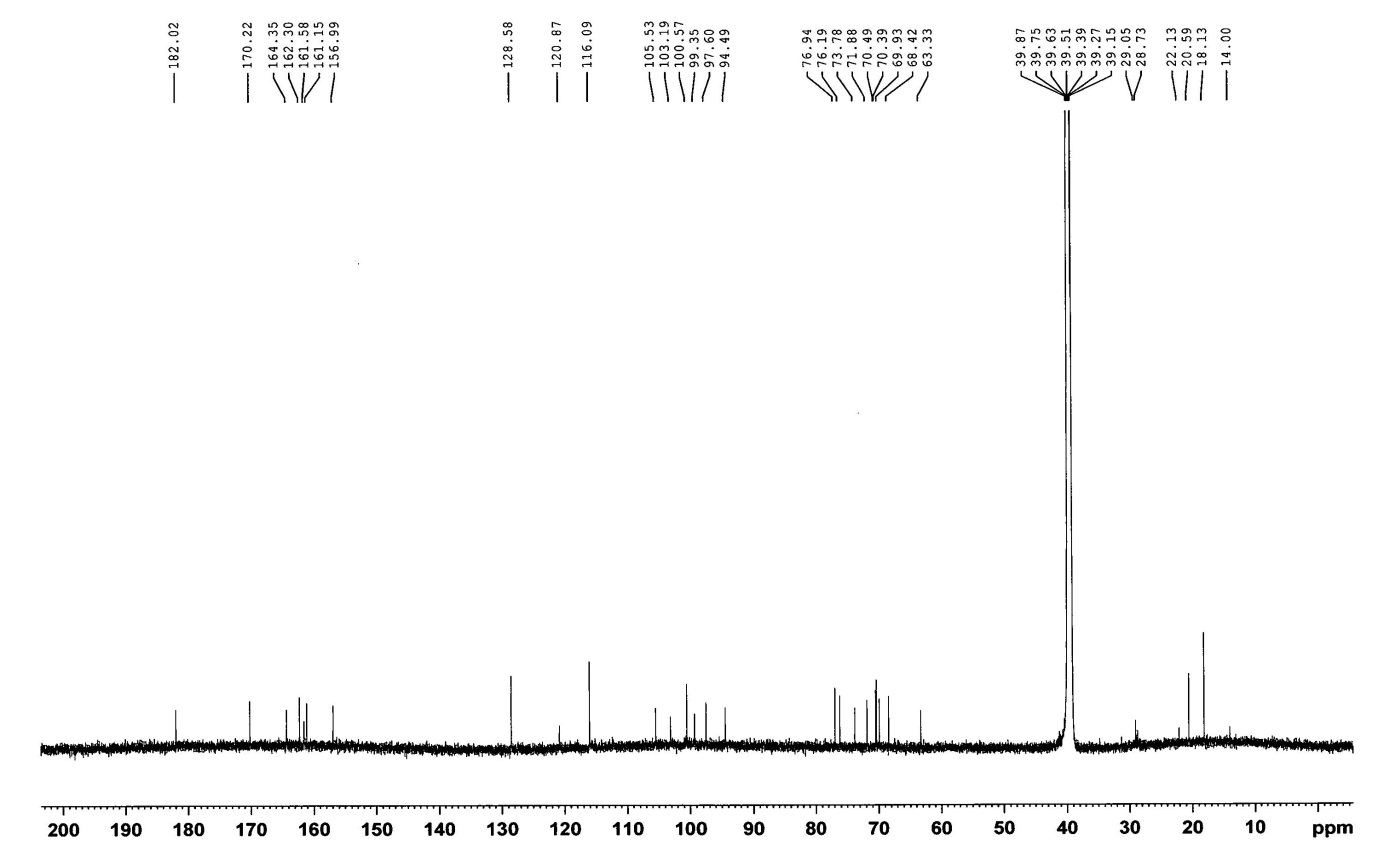


**Figure S13**. ^13^C NMR spectrum of compound **2** (DMSO-*d*_6_, 176 MHz).


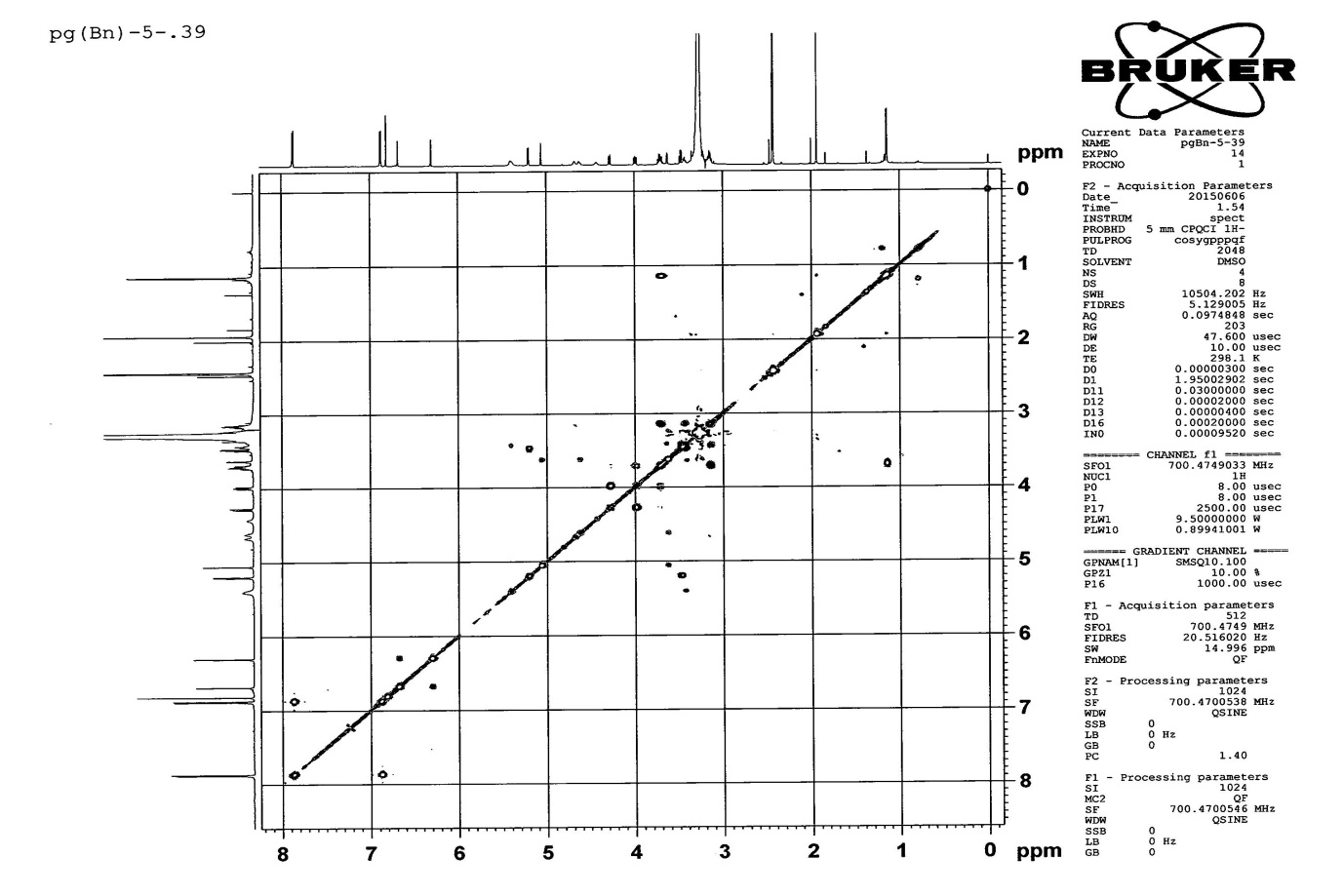


**Figure S14**. ^1^H-^1^H COSY spectrum of compound **2** (DMSO-*d*_6_, 700 MHz).


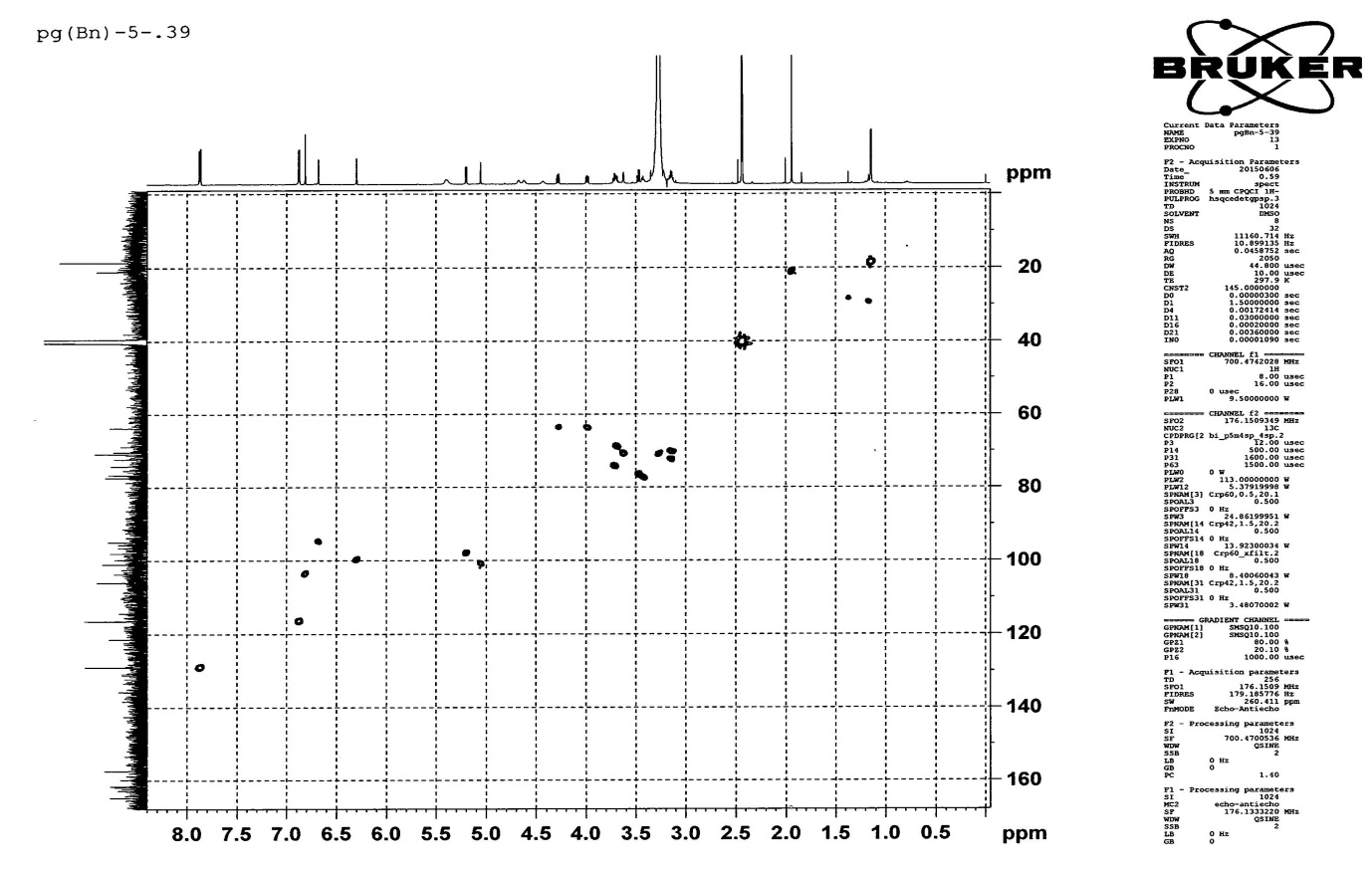


**Figure S15**. HSQC spectrum of compound **2** (DMSO-*d*_6_, 700 MHz).

**
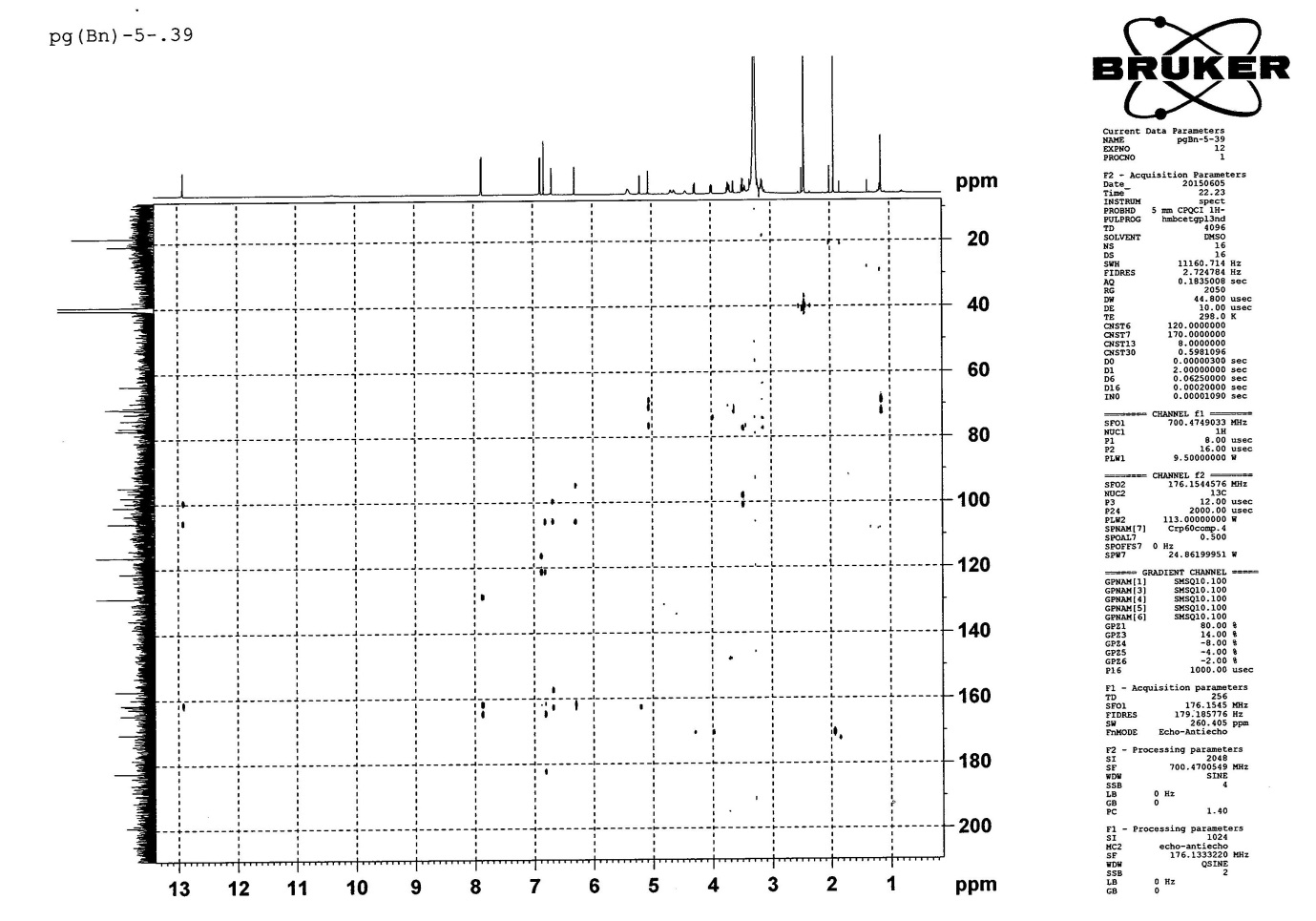
**

**Figure S16**. HMBC spectrum of compound **2** (DMSO-*d*_6_, 700 MHz).
